# Prevalence and Clinical Significance of Drug–Drug and Drug–Dietary Supplement Interactions among Patients Admitted for Cardiothoracic Surgery in Greece

**DOI:** 10.3390/pharmaceutics13020239

**Published:** 2021-02-09

**Authors:** Marios Spanakis, Maria Melissourgaki, George Lazopoulos, Athina E. Patelarou, Evridiki Patelarou

**Affiliations:** 1 Department of Nursing, Faculty of Health Sciences, Hellenic Mediterranean University, GR-71004 Heraklion, Greece; apatelarou@hmu.gr (A.E.P.); epatelarou@hmu.gr (E.P.); 2 Computational Biomedicine Laboratory, Institute of Computer Science, Foundation for Research & Technology-Hellas (FORTH), GR-70013 Heraklion, Greece; 3 Cardiothoracic Surgery Division, University Hospital of Heraklion, GR-71500 Heraklion, Greece; melimargr@yahoo.gr (M.M.); g.lazopoulos@uoc.gr (G.L.); 4 School of Medicine, University of Crete, GR-71500 Heraklion, Greece

**Keywords:** drug–drug interactions, pharmacokinetic interactions, pharmacodynamic interactions, drug–herb interactions, drug–food interactions, cardiovascular disease, cardiothoracic surgery

## Abstract

Background: Drug interactions represent a major issue in clinical settings, especially for critically ill patients such as those with cardiovascular disease (CVD) who require cardiothoracic surgery (CTS) and receive a high number of different medications. Methods: A cross-sectional study aimed at evaluating the exposure and clinical significance of drug–drug (DDIs) and drug–dietary supplement interactions (DDSIs) in patients admitted for CTS in the University Hospital of Crete Greece. DDIs were evaluated regarding underlying pharmacological mechanisms upon admission, preoperation, postoperation, and discharge from CTS clinic. Additionally, upon admission, the use of dietary supplements (DSs) and if patients had informed their treating physician that they were using these were recorded with subsequent analysis of potential DDSIs with prescribed medications. Results: The study employed 76 patients who were admitted for CTS and accepted to participate. Overall, 166 unique DDIs were identified, with 32% of them being related to pharmacokinetic (PK) processes and the rest (68%) were related to possible alterations of pharmacodynamic (PD) action. CVD medications and drugs for central nervous system disorders were the most frequently interacting medications. In total, 12% of the identified DDIs were of serious clinical significance. The frequency of PK-DDIs was higher during admission and discharge, whereas PD-DDIs were mainly recorded during pre- and postoperation periods. Regarding DS usage, 60% of patients were using DSs and perceived them as safe, and the majority had not informed their treating physician of this or sought out medical advice. Analysis of medical records showed 30 potential combinations with prescribed medications that could lead in DDSIs due to modulation of PK or PD processes, and grapefruit juice consumption was involved in 38% of them. Conclusions: An increased burden of DDIs and DDSIs was identified mostly upon admission for patients in CTS clinics in Greece. Healthcare providers, especially prescribing physicians in Greece, should always take into consideration the possibility of DDIs and the likely use of DS products by patients to promote their well-being; this should only be undertaken after receiving medical advice and an evidenced-based evaluation.

## 1. Introduction

Drug–drug interactions (DDIs) can modulate the clinical outcome of coadministered drugs by altering their main or secondary pharmacological actions and ultimately lead to the development of adverse drug reactions (ADRs). This may result in impaired health in patient, complicating the treatment of a pathological condition and possible prolonging hospitalization [1,2]. The underlying pharmacological mechanisms of DDIs are related either to synergistic or competitive actions on specific receptors (pharmacodynamic interactions, PD-DDIs) or by altering pharmacokinetic (PK) processes of absorption, distribution, metabolism, and elimination (ADME), thus leading to PK-DDIs. DDIs can be evaluated and categorized in relation to the severity of the ADRs and the resulting clinical outcome [3]. Regarding the frequency and the severity of DDIs, additional factors that seem to play a role are patient age group, number of administered drugs, comorbidities, pharmacogenomic and pharmacogenetic characteristics as well as dietary and lifestyle habits [4,5,6,7,8].

Patients admitted for cardiothoracic surgery (CTS) represent a special patient population group that receives an increased number of medications and needs advanced healthcare provision during hospitalization [9]. Most of them are diagnosed with cardiovascular diseases (CVDs) along with several other comorbidities such as diabetes, obesity, chronic obstructive pulmonary disease, central nervous system (CNS) disorders, dyslipidemias, etc. [10]. CVD refers to a group of disorders such as coronary artery disease, cerebrovascular disease, peripheral vascular disease, and other conditions with the subsequent development of heart failure and cardiac arrhythmias which require medical treatment and, in cases of disease progression, surgical interventions [11]. Some of the most frequent CTS interventions are to coronary artery bypass graft surgery (CABG), surgical aortic valve replacement (SAVR), mitral valve prolapse (MVPl), carotid endarterectomy (CEA), Bentall surgery for aortic root replacement, abdominal aortic aneurysm (AAA), type B aortic dissection, heart injury, postoperative hernia, and myxoma L atrium. All these surgical procedures require the admission of the patient in the CTS clinic, and preoperative preparation and postsurgery monitoring of patients’ health statuses, both of which require specialized medical interventions and discharge [12].

Prior to any CTS procedure, these patients are often advised to made essential changes in their dietary and lifestyle habits to improve their well-being and to prevent disease progression [13]. In this effort, patients often incorporate dietary supplements (DSs) such as nutraceuticals, dietary food supplements or herbal products in their daily diet [14]. These attitudes are occasionally supported by other factors, such as self-medication beliefs, misguidance from limited or misinterpreted scientific data, sociological factors and marketing advertisements, dissatisfaction with conventional medication, and/or lack of communication with healthcare providers [15,16].

Until today, several studies regarding CVD patients and DDIs or DDSIs focused on potential ADRs related to administered medication during hospitalization or with DS consumption. It is estimated that approximately 3–5% of DDIs among CVD patients in hospital units are serious while 60–70% of them are of major clinical significance and most of them can result in ADRs [17,18]. Regarding DS usage, several studies have demonstrated that up to 60% of CVD patients may use DSs in their daily diets to promote their well-being without informing their healthcare providers, putting themselves at risk of a significant DDSI due to concurrent administration of interacting medications [19,20,21]. Concerning Greece, except for prescriptions dispensed in pharmacies [22], there are no data available from clinical studies about potential DDIs during hospitalization (which requires an increased number of medications to be administered) as well as the use of DSs and their potential drug interactions. The aim of this study was to record and analyze the prevalence of DDIs in different time points during hospitalization of patients admitted for CTS and examine possible pharmacological mechanisms involved. Moreover, the study tried to record the habitually use of DSs and determine if it is based on medical advice as well as, to identify and estimate the exposure in potential DDSIs with prescribed medications for these patients.

## 2. Materials and Methods

### 2.1. Study Design and Ethics Approval

The cross-sectional study was conducted over a 6-month period in the division of Cardiothoracic Surgery Division of University Hospital of Heraklion in Greece. The study was carried out following the rules of the Declaration of Helsinki of 1975, as revised in 2013, and it was complied with the General Data Protection Regulation (GDPR) and approved by the hospital’s ethics committee. Moreover, the study followed guidelines for reporting observational studies (Strengthening the Reporting of Observational Studies in Epidemiology—STROBE) and evidence-based medicine approaches [23]. The STROBE information is provided in Table 1.

All patients admitted in the CTS clinic were given an information brochure of the study and included to participate after they singed the informed consent form. All participants were adults (18–65 years old) or elderly (>65 years old) who were admitted for CTS in the clinic, administered more than two medications, and hospitalized for more than two days. Patients on short-term hospitalization or with <2 drugs, those who did not understand the terms of participation and consent, and cases of readmission causally related to previous cardiac surgery were reasons for patients to be excluded from the study. For each participant, upon admission and during hospitalization, the primary investigator completed a questionnaire regarding demographics, comorbidities, clinical data during hospitalization, and medication regimens at four discrete time points: (i) admission (patients enter the clinic), (ii) preoperation (patients enter the preoperation phase to be prepared for surgery), (iii) postoperation (patients are monitored to recover from surgery), (iv) discharge (patients are stable and exit the clinic). Moreover, for the positive answers of DS usage, the participants state the DSs that they are using, if they had informed their treating physician (YES/NO), if they use it as part of self-medication habits (YES/NO), if medical advice was requested (or provided) prior to use (YES/NO), and the reasons of using DSs (recording the statement verbatim). All data were collected and analyzed anonymously, and no interventions were made regarding healthcare provision during hospitalization.

### 2.2. Evaluation of Drug Interactions with Coadministered Medications and Interactions with Dietary Supplements

Drug combinations that may result in DDIs and DDSIs were detected using available online drug interaction checker tools (Medscape and Drugs.com). The categorization and the clinical significance of the identified interactions were further assessed based on the availability of scientific data from the literature which provide sufficient evidence of the underlying biological mechanisms and the outcomes. The evaluation was based on different levels of evidence that describe significance, such as theoretical mechanisms, in silico/ in vitro/in vivo data, clinical studies, expert opinion reviews, meta-analysis, information encompassed in the drug’s label, summary of product characteristics (SmPC) or in reports from regulation between interacting drugs or drugs from the same pharmacological group. The clinical significance of the interactions in this study is represented as *“Serious–Use alternative”*, *“Use with caution-Monitor”* and “*Moderate-Minor”*. A similar analysis regarding the clinical significance from drug interactions with dietary supplements was followed [24,25].

## 3. Results

### 3.1. Patients’ Demographics, CTS Diagnoses, Comorbidities, and Clinical Data during Hospitalization

**Figure 1:**
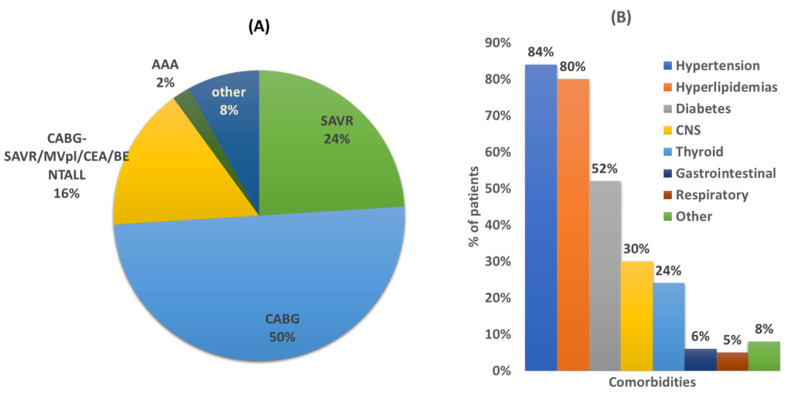
(**A**) Diagnosis for CTS surgery. Coronary artery bypass graft (CABG) surgery; surgical aortic valve replacement (SAVR); mitral valve prolapse (MVPl); carotid endarterectomy (CEA); abdominal aortic aneurysm (AAA); Bentall: aortic root replacement surgical procedure. (**B**) Comorbidities of studied population. CNS: central nervous system. Other comorbidities refer to rheumatoid arthritis, cancer, prostatic hyperplasia, sleep apnea, hepatitis C, chronic kidney failure, and scleroderma.

Over the 6-month period, 76 (46 male, 30 female) of the total 80 patients admitted in the CTS unit for scheduled (86%, *n* = 65) or urgent (14%, *n* = 11) surgery, met the study criteria and were accepted for participation in the study. Table 2 summarizes their demographics, clinical characteristics, and social habits. The diagnosis for CTS admittance for most of the cases were CABG surgery (*n* = 38, 50%) (Figure 1A). The average age of patients was 66 years old (min 37, max 85), and the mean body mass index (BMI) was 29.9 (min 17.1, max 45.4) with an average number of five comorbidities. The most prevalent comorbidities were hypertension (84%), hyperlipidemias (80%), and diabetes (52%) (Figure 1B). Regarding social habits, there was a high prevalence of smokers or ex-smokers (more than two months smoking cessation) as well as social drinkers (Table 2).

The study also recorded the routine lab test results regarding biomarkers during hospitalization. All patients appear to follow the expected clinical profile as surgical patients (see Supplementary file). The mean clinical values regarding heart rate, creatinine, potassium (K^+^), sodium (Na^+^), international normalized ratio (INR), and blood glucose seem to follow the expected clinical course with small variations from the anticipated values, which are related to the surgery and modification in medications (see Supplementary file).

### 3.2. Medications Administered at Different Time Points

**Figure 2:**
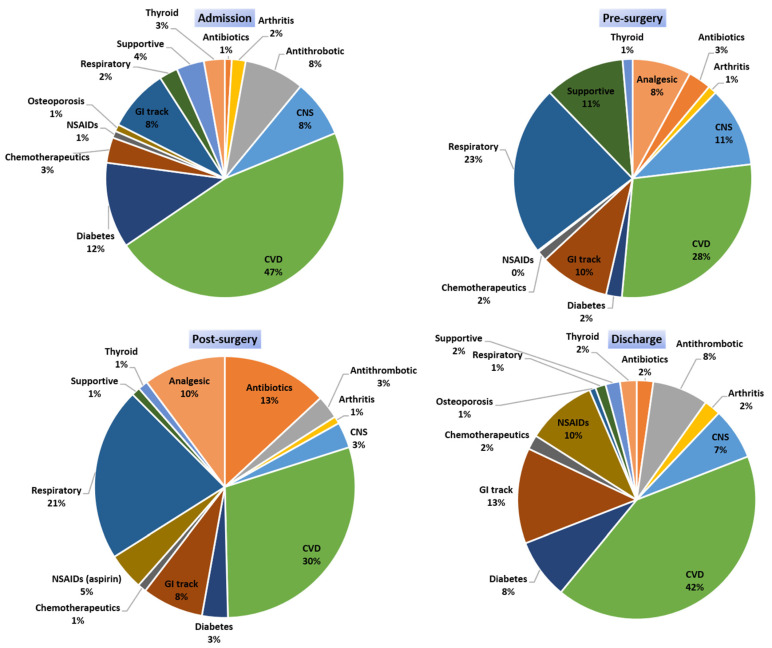
Medication categories that were administered at the different time points of the study. (CVD: cardiovascular disease medications; CNS: central nervous system; GI track: H2 blockers and PPIs: proton-pump inhibitors; antacids NSAIDs: nonsteroidal anti-inflammatory drugs; supportive: supplementary products such as electrolytes, lactulose, ferrous, etc.).

The drug categories that were administered to patients in each period (admission, preoperative, postoperative, and discharge) are presented in Figure 2. Patients during their hospitalizations, and according to the healthcare provision that provided them, received medications from several drug categories such as analgesics, antibiotics; antithrombotic agents, arthritis (immunosuppressants); chemotherapeutics; CNS disorders; CVD; diabetes (insulin and/or antidiabetics); drugs for gastrointestinal (GI) track-related disorders; anti-inflammatory and antirheumatic products such as nonsteroidal anti-inflammatory drugs (NSAIDs) and drugs for treatment of bone diseases (i.e., osteoporosis); thyroid therapy drugs; medications for respiratory system; supportive medications such as drugs for constipation, antiemetics, mineral supplements, and vitamins. The average number of drugs (± standard deviation) administered per time point was 7 ± 3 (min 1, max 18) upon admission, 13 ± 4 (min 4, max 16) during preoperation, 14 ± 2 (min 7, max 18) in postoperation, and 7 ± 3 (min 3, max 16) in discharge. CVD medications were the most frequently administered category at all time points. Respiratory medications were mostly administered upon admission and preoperation. Analgesics and opioids for pain management were similarly administered before and after surgery. Central nervous system (CNS) medications, except those prescribed and recorded upon admission, were mainly administered in preoperation period. Antibiotics were mostly administered in the postsurgery period. Diabetes medications recorded upon admission were reduced before surgery and reintroduced when the patient was stable and able to consume food in postoperation period or discharge. Proton-pump inhibitors (PPIs) and H-2 histamine antagonists were administered during all time periods. NSAIDs (including aspirin) were stopped preoperation and reintroduced upon discharge. Medications that are prescribed for other comorbidities were administrated continuously along with supportive products such as lactulose, electrolytes, ferrous, etc., which were administered based on each patient’s needs.

### 3.3. DDIs Identified and Correlation with Administered Medications

**Figure 3:**
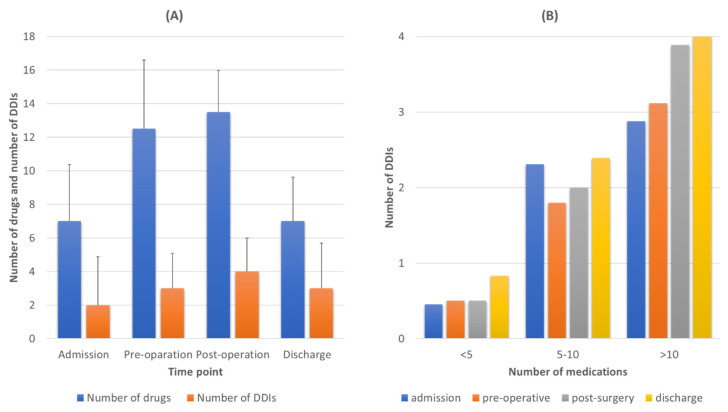
(**A**) Average number of medications administered, and average number of drug–drug interactions (DDIs) detected for each time point of the study. (**B**) Correlation between the number of medications and number of DDIs detected for each time point.

Based on the number of medications administered, there was an important number of DDIs during all time points (Figure 3A). Overall, there were 688 cases of DDIs from 166 different drug combinations of various clinical significance (see Supplementary file for full list). An average value of 3 (min 0, max 18) DDIs per patient was observed during admission and the preoperative period (min = 0, max = 10), four DDIs per patient in the postoperative period (min = 0, max = 12), and three DDIs during discharge (min = 0, max = 17). There was a positive trend of an increased number of DDIs during hospitalization related to the increased number of medications administered, especially in the postoperative period comparing to other time points (Figure 3A). There was a positive trend between number of medications administered and number of DDIs detected, revealing that as number of medications rises, the risk for DDIs also increases regardless of the time point (admission, pre- or postoperation and discharge) (Figure 3B). The percentage of patients exposed to one, two or three, or four or more clinically significant DDIs is presented in the supplementary file.

### 3.4. Pharmacological Mechanisms and Clinical Significance of the Identified DDIs

**Figure 4:**
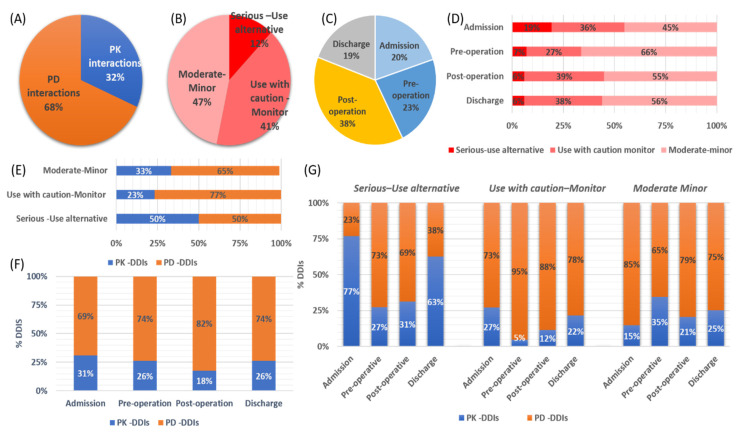
(**A**) Pharmacological mechanisms. (**B**) clinical significance of the identified drug–drug interactions (DDIs). (**C**) Time points of the DDIs. (**D**) DDI risk categories distributed over the four time points. (**E**) Clinical significance and pharmacological mechanisms involved. (**F**) Pharmacological mechanisms over time points for the DDIs. (**G**) Pharmacological mechanisms, time point, and clinical significance of the recorded DDIs (PK-DDIs: pharmacokinetic DDIs, PD-DDIs: phamacodynamic DDIs).

**Figure 5:**
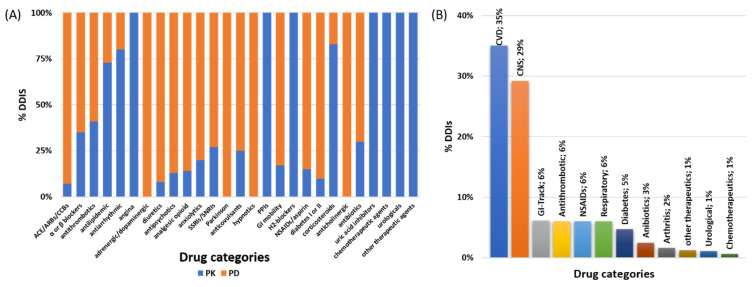
(**A**) PK-DDIS and PD-DDI distribution among medications and (**B**) overall drug categories that were involved in DDIs for the current study. (ACE: angiotensin-converting enzyme inhibitors; ARBs: angiotensin receptor blockers; CCBs: Ca^2+^ channel blockers; GI: gastrointestinal system; NSAIDs: nonsteroidal anti-inflammatory drugs).

The involved pharmacological mechanisms of the identified DDIs were related to either PK (32%) or PD (68%) processes (Figure 4A). Regarding their clinical significance, 12% of the identified DDIs were characterized as a “Serious–Use alternative”, with 41% of them as “Use with caution-Monitor”, and the remaining 47% were of “Moderate -Minor” significance (Figure 4B). DDIs seemed to be recorded to similar extents at all time points of hospitalization (Figure 4C). PD-DDIs seemed to be mostly of “Moderate-Minor” clinical significance, or the combination could be administered but needed to be “Use with caution-Monitor”. Although PD-DDIs were recorded at all time points, the highest prevalence of them was found in the postoperation period (Figure 4D). On the other hand, the identified PK-DDIs mostly referred to cases of “serious-use alternative” clinical significance, and most of them were observed upon admission and in discharge (Figure 4E,F). Table 3 shows the recorded PK-DDIs and PD-DDIs of serious clinical significance along with the most frequent cases for the rest of DDIs (see Supplementary file for the full list). PK-DDIs were related to administration of α- and β-blocker, antithrombotic, antilipidemic, antiarrhythmic, angina, antipsychotics, anxiolytics, proton-pump inhibitors (PPIs), H2-blockers, and corticosteroids medications. Particularly, the 10 most frequently used medications related to PK-DDIs were acenocoumarol, budesonide, esomeprazole, alprazolam, clopidogrel, amiodarone, ranolazine, haloperidol, simvastatin, metoprolol, and aspirin. PD-DDIs were mostly related to administration of angiotensin-converting enzyme (ACE) inhibitors, angiotensin receptor blockers (ARBs), and calcium channel blockers (CCBs), diuretics, adrenergic/dopaminergic agents, antipsychotics, anxiolytics, serotonin, and norepinephrine reuptake inhibitors (SSRIs/SSNRIs), NSAIDs, and anticholinergics (Figure 5A). Regarding the 10 most frequent medications that were related to PD-DDIs, furosemide, aspirin, enoxaparin, ceftriaxone, morphine, insulin, acenocoumarol, quetiapine, perindopril, metoprolol, and bromazepam were most frequently recorded. Overall, drugs for CVD and CNS were the most prevalent for DDIs (Figure 5B) in the current study.

### 3.5. Dietary Supplements, Reasons for Use, and Identified DDSIs

**Figure 6:**
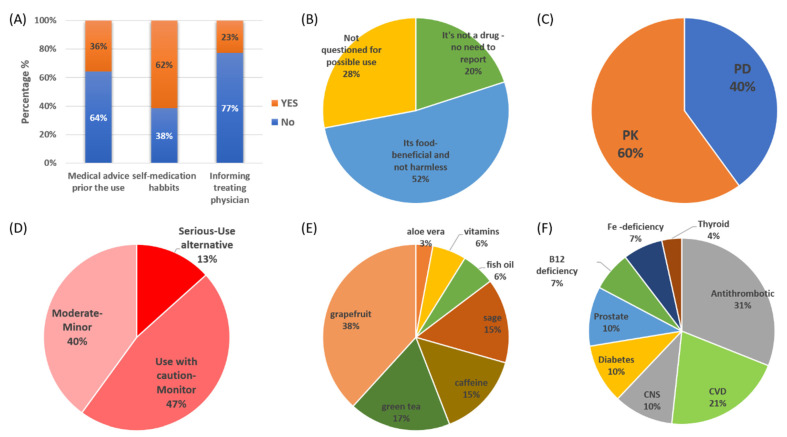
(**A**) Attitudes and (**B**) perceptions regarding the use of DSs from patients in the study. Pharmacological mechanisms (**C**) and clinical significance (**D**) of the identified interactions between prescribed medications and DSs in the study. (**E**) DS categories and (**F**) drug categories identified with potential interactions (CNS: central nervous system; CVD: cardiovascular drugs, including lipid-lowering therapies).

DSs were used by 60% of the participants in the study (*n* = 46). As for food products, 56% of the patients stated that they drink mixed commercial fruit juices while 90% of the cohort drink coffee daily. The consumption of DSs refers to aloe or senna laxative products (18%, *n* = 8), green tea (30%, *n* = 14), sage/salvia/chamomile (36%, *n* = 17), grapefruit juice (26%, *n* = 12), and fish oil/vitamins (20%, *n* = 9). From the 46 patients, only seven (*n* = 7, YES, 15%) stated that they used them after medical advice or informed their treating physician. The medical advice regarded consumption of tea and sage (*n* = 3), vitamin products (*n* = 3), and fish oil (*n* = 1). Most of the DS users stated that they did not seek medical advice (NO, 64%) or inform their treating physician (NO, 77%) and they use them as part of their self-medication habits (YES, 62%) (Figure 6A). The reasons for use without medical advice for the 39 patients, were stated to be *“it’s not a drug no need to report”* (*n* = 8, 20%), *“it is food-beneficial and not harmless”* (*n* = 20, 52%), and *“not questioned for DS use”* (*n* = 11, 28%) (Figure 6B).

The analysis of data regarding prescribed medications, as recorded upon admission, along with DS usage revealed 30 unique cases of potential DDSIs (62 DDSIs in total). The 30 combinations and their significances are presented in Table 4. For all these cases, available scientific data in the literature support the potential for interaction. The pharmacological mechanisms of the DDSIs were related in 60% of them with modulation of PK processes and the rest 40% potential interactions through PD mechanisms (Figure 6C). The severity of potential DDSIs were categorized as “Serious-Use alternative” in 13% of the identified cases, “Use with caution-Monitor” in 47%, and “Moderate-Minor” for the remaining 40% (Figure 6C). DSs that were more likely to present potential interactions were grapefruit juice (38%), green tea (17%), and sage (15%) (Figure 6D). The frequent consumption of grapefruit as a DS stands out regarding DDSIs in the cohort. Caffeine consumption was related to potential impact on the absorption of levothyroxine (15%). Potential clinically significant DDSIs were found for antithrombotic agents (31%), CVD (21%), CNS medications (13%), and for diabetes (10%) (Figure 6F).

## 4. Discussion

DDIs represent a serious clinical issue in healthcare provision, especially for critically ill patients. Assessment of potential DDIs not only reveals prescription errors but also assists the healthcare provision by informing medical teams on what precautions should be made for administration of specific medications. Polypharmacy, comorbidities, DS usage, hospitalization period, and patients’ health statuses are contributing factors for the appearance of DDIs which could lead to ADRs, prolonged hospitalization, increased healthcare costs, and reduction in patients’ quality of life [26]. This study recorded and analyzed the prevalence of DDIs at four discrete time points of hospitalization for patients admitted to hospital for CTS procedures. CTS patients in our study have clinical characteristics that place them in a high-risk group for DDIs, such as an average age of 66 years, being admitted for CTS (mainly for CABG surgery), comorbidities (average of five), administered a high number of medications (average of 10 drugs), and hospitalized for approximately 10 days [6,7].

In this study, a positive trend was observed between number of medications and prevalence of DDIs (Figure 3). Although most of the identified DDIs were found to be of “Use-with caution-Monitor” or of “Moderate-Minor” clinical significance, 12% of the identified DDIs were considered to be of “Serious-Use alterative”, indicating that a better evaluation of medication regimens should be considered for these patients especially (Figure 4) [7,17,27,28,29]. The highest occurrence of DDIs was observed during hospitalization and especially in the postoperation period where the number of administered medications rose [1]. Regarding the pharmacological mechanisms involved, most of DDIs described as PD-DDIs were related to synergistic effects of coadministered medications for all time points except admission. PD-DDIs are usually observed in similar studies and their clinical considerations are based on the risk–benefit analysis by the medical team [30]. The identified PK interactions in our cohort were mostly related with inhibition of mediated metabolism from cytochrome P450 (i.e., CYP1A1, CYP1A2, CYP2C19, CYP2D6, and CYP3A4) and transporter inhibition (i.e., organic anion transporter B1, OATB1 or organic cation transporter 2, OCT2). The inhibition of these PK processes can result in modulation of drug clearance and renal elimination and thus in ADRs from increased concentrations for the interacting drugs. Although PK-DDIs were recorded to a lesser extent than PD-DDIs, they were of high prevalence upon admission and discharge and most of PK-DDIs were characterized as “Serious-Use alternative” clinical significance (Figure 4). The same trend was observed for medications upon discharge where patients return to their initial therapeutic regimens. This is probably due to errors during prescription or lack of awareness for PK-DDIs, which have been observed in the past in prescribing physicians in Greece [22]. Overall, the prevalence of DDIs, especially the 12% of those of “Serious-Use alternative”, is comparative with other studies and reports of hospitalized patients [7,9,17,31,32].

The analysis of the medication regimens during hospitalization revealed that most of the medications related to DDIs were drugs for CVD such as metoprolol, amiodarone, ranolazine, perindopril, furosemide; drugs acting on CNS such as alprazolam, bromazepam, haloperidol, morphine, and quetiapine; antithrombotic agents such as acenocoumarol, enoxaparin, and clopidogrel; antilipidemic agents and especially statins; insulin, PPIs such as esonemprazole; antibiotics such as ciprofloxacin or ceftriaxone; aspirin. Our results indicate that these drugs should be administered under constant consideration of potential and clinically significant DDIs. Antiarrhythmics such as amiodarone, a known CYP2D6 inhibitor, were mostly involved in PK-DDIs with β-blockers, statins, and analgesics such as tramadol and morphine. Diuretics (i.e., furosemide) were related to potential PD-DDIs and synergistic or antagonistic effects regarding potassium levels that should be monitored [33]. Drugs for angina treatment were mostly related to PK-DDIs when coadministered with β-blockers, statins, diabetes medications, and opioids. Especially for ranolazine, interacting combinations with metoprolol, carvedilol, metformin, and statins were recorded. Ranolazine can inhibit CYP-mediated metabolism (CYP3A4) and/or transport proteins and thus lead to increased plasma levels of drug substrates and potential ADRs such as lactic acidosis when coadministered with metformin or myopathy and rhabdomyolysis with simvastatin [34,35]. Ca^2+^ blockers, such as amlodipine, can interact with statins or metformin, which could lead to increased PD action, whereas combinations of amlodipine with β-blockers or ARBs (i.e., irvesartan) should be used only under risk–benefit analysis and potassium level monitoring [32]. Concerning antithrombotic agents, DDIs that potentiate anticoagulation or antiplatelet activity due to modulation of metabolism or synergistic effects were also recorded. Generally, DDIs of antiplatelet agents with PPIs, Ca^2+^ blockers, statins, or NSAIDs as well as clinically relevant DDIs of coumarin analogues with drugs that inhibit CYP-mediated metabolism (PPIs, amiodarone etc.) or NSAIDs and SSRIs that can increase risk of bleeding have been well described and these combinations should always be used with caution and monitored [36,37]. The current study also showed that CTS patients tend to receive an increased number of CNS drugs such as antipsychotics, anxiolytics, and antidepressants to deal with their anxiety and stress. In our study, the most frequent drugs related to DDIs were haloperidol and alprazolam, which can result in synergistic PD effects such as sedation when other CNS-acting drugs are co-administered (i.e., analgesics). Generally, from our results, CNS drug combinations were considered as DDIs due to their synergistic effects. Sometimes, combinations of CNS drugs are proposed in a risk–benefit analysis for patients that do not respond to monotherapy. However, as this study considers the target patient population, the combination of quetiapine-haloperidol was characterized as “Serious-Use alternative” due to the risk for QT prolongation, which can result in arrhythmia, a significant complication for CTS patients. Finally, another important drug category in our study that is often associated with DDIs was antibiotics. In this study, ceftriaxone or tazobactam could be related to increased PD action of anticoagulants; thus, these coadministrations were categorized as “Use with caution-Monitor” for the resulting DDIs [9].

In this work, the issue of drug interactions was also approached from another point of view that of the potential interaction of prescribed medications with dietary products (DDSIs). Many of the patients in our cohort were habitually using DSs without considering the risks of potential interactions with their medications and were neglecting informing their treating physicians (Figure 6, Table 4) [38]. DS users in this study were found to often consume herbal teas (green tea, sage) as traditional DSs that promote well-being, aloe vera or senna for constipation issues, grapefruit juice for weight control management, and fish oil as part of their lipid-lowering diet [39,40,41,42]. Generally, the results are comparative with previously published works regarding the prevalence of regular use of DSs among CVD patients, especially in preoperative periods [19,43,44,45,46]. It can be argued that, although scientific evidence is available, it is not disseminated sufficiently from healthcare providers to patients [24]. For example, the identified and clinically significant DDSIs (38%) in this study were related to grapefruit consumption which is known to interact with several medications [47,48,49]. In our study, grapefruit interactions were found for CVD drugs (ranolazine, statins, and diuretics), antithrombotic agents, and prostate hyperplasia medications. Grapefruit is widely used for weight loss, especially among obese patients, although one of the most typical examples of drug–grapefruit interactions is with statins that may lead to clinical significant ADRs such as rhabdomyolysis [50,51]. The underlying pharmacological interaction is due to the mechanism-based inhibition of CYP enzymes (i.e., CYP3A isoforms) from furanocoumarins found in grapefruit or inhibition of transporter proteins from other constituents. This leads to increased bioavailability during the absorption phase along with reduction in intrinsic clearance, mainly for orally administered drugs that undergo substantial first-pass effect and liver metabolism from CYP3A [49,52]. Tea products (green tea, sage) are popular in Greece and thus the increased usage among patients was expected; however, 32% of the identified interactions were referring to tea and sage products due to their capabilities to modulate the absorption of iron and folic acid supplements and in some cases interact with CVD medications [53,54]. Aloe vera has demonstrated hypoglycemic effects and may enhance the pharmacological activity of antidiabetic agents or insulin administration due to the potential PD interactions from constituents present in aloe vera and can synergistically modulate insulin sensitivity in tissues [55,56]. Fish oil, due to the high content in omega-3 fatty acids, is often used as a DS among patients but with an extensive debate regarding its benefits in CVD and experimental data suggesting that omega-3-fatty acids may reduce thrombin generation and plasma levels of fibrinogen, prothrombin, and coagulation factors, potentiating the pharmacologic effects of anticoagulants or antiplatelet drugs as well as aspirin [57,58]. Finally, regarding food habits, the frequent consumption of fruit juices (56%) and coffee (90%) cannot go unnoticed. Although specific interactions of fruit juices were not identified in the present study, scientific data suggest that some commercially available fruit juices can be related to PK interactions with drugs [59]. The mechanism is suggested to be due to the inhibitory effects of constituents of fruit juices on CYP3A-mediated metabolism and/or transport through OATP for several drugs [59,60]. Coffee consumption has been extensively studied in CVD patients and generally reasonable caffeine intake is not associated with increased risks [61]. Caffeine, however, has been proposed to reduce gastrointestinal absorption of levothyroxine [62]. Finally, the potential interaction between B12 with aspirin or proton-pump inhibitors (esomeprazole) which may lead to poor B12 absorption and deficiency was also recorded [63].

The evaluation of patients’ clinical statuses is important to help minimize and prevent any adverse events such as DDIs [64]. The lab test results, and patients’ clinical statuses monitored in our study do not seem to show a clear causal relationship with the identified DDIs (especially for PD-DDIs); however, the aggravating role of DDIs cannot be ruled out. Variations in heart rate and liver enzymes in postoperative period are expected for CTS patients, usually attributed in the surgery or to some extent in the modification of administered medications. Atrial fibrillation or sinus tachycardia are often observed in postoperative patients due to pre-existing arrhythmia or stimulus of the sympathetic nerve [65,66]. Moreover, atrial fibrillation tachycardia, etc., are common reported adverse events that occur frequently after cardiac surgery in 11–40% of patients who underwent CABG and more than 50% of patients with heart valve replacements [65]. Additionally, rises in hepatic enzymes after heart surgery is common in 10% of patients. Temporary but not enduring alterations of hepatic enzymes are often observed, attributed to several reasons which include complications that may be a consequence of the operation, pathological conditions, and also the administered medications [67].

In hospital settings, patient safety issues are a major concern. To minimize the risk of medication errors, tools such as electronic decision support systems that allow the prospective evaluation of interactions should be used in clinical practice when making decisions to optimize patient safety [68]. Some of the advantages of using electronic healthcare tools for DDIs in clinical practice are the collection of information that facilitates the identification of the main DDIs (or DDSIs) with relative clinical risk and prioritizes the necessary actions to improve healthcare as well as improve communication between healthcare providers and patients (in the case of DDSIs) [24,68,69]. Prevention of DDIs and ADRs before they occur is an urgent need even for the less obvious ADRs and requires constant vigilance, accurate understanding of the etiology, and correct diagnosis. A thoughtful consideration from a specialized medical team that follows detailed evidence-based clinical guidelines regarding drug administration is beneficial for patients, reducing medication errors and improving the incidence of DDIs [70]. Continuous education is considered necessary to enhance awareness regarding PK/PD mechanisms of DDIs, ensuring the safety and efficacy of administered treatment especially in cases of critically ill patients such as CTS patients. In this way, healthcare professionals will develop a safe, effective, and personalized drug treatment plan for inpatients, preventing and minimizing the potential risk and incidence of treatment-related problems, with a view to optimizing treatment and patient safety.

The strengths of this work as an observational study are that it tried to record and analyze all types of interactions for a high-risk group such as CTS patients, and approached the prevalence of DDIs and DDSIs using more than one drug interaction checker as well as available literature. The use of additional checkers for analysis of DDIs retracts previous reported limitations regarding the number of DDIs that could be recognized. Moreover, available checkers provide different methods of DDI characterization or different clinical severity classifications, complicating the comparison among studies [9,17,18,22]. The performance of the available checkers has been evaluated previously regarding their capability of returning similar or at least comparable results [71]. Therefore, incorporation of data from the available literature allowed us to analyze cases that were not listed in both checkers or cases of different results and make an overall better evaluation of clinical significance of identified interactions. One of the limitations of the study is the small study sample due to the study duration (6 months) in one clinic (approximately 2–3 preoperative patients per week) which is due to the location of the study in Heraklion prefecture, which has a population of approximately 300,000 people. Additionally, the study focused on a subgroup of CVD patients—those who required CTS due to disease progression or diagnosis, which did not favor the registration of a larger cohort group (perhaps with additional hospitals or all the CVD patients). However, most of them (94% of admitted patients) accepted to participate, thus it can be argued that they were a representative study sample regarding CVD patients that undergo CTS in Greece, especially regarding the usual medication regimens that are administered to them. Especially for the DDSIs, the incorporation of larger and different patient cohorts in future studies is needed to better describe the prevalence of DDSIs in the Greek population. Another limitation of the study was that even though the severity of potential DDIs was identified, any occurrences of ADRs attributed to DDIs or DS consumption could not be recorded or causally related due to the absence of sufficient evidence during hospitalization in the clinic and lack of relative information in patients’ medical records. Additionally, during their hospitalization, DS consumption was avoided. Overall, despite its limitations, the current study—considering Greece for the first time—described potential and clinically significant interactions among critically ill hospitalized patients. In the context of relative initiatives from the EU for its member states, these studies can provide valuable information regarding pharmacovigilance and drug safety monitoring not only for healthcare providers in Greece, but also for the EU healthcare ecosystem in general [72].

## 5. Conclusions

The present cross-sectional study explored the prevalence of DDIs among patients during hospitalization in a CTS hospital clinic in Greece and their habitually use of DS products that could lead to DDSIs. Our results indicate a high exposure of DDIs and DDSIs for patients that undergo CTS surgery in Greece, with 12% of the identified DDIs to be of serious clinical significance, and that alternative combinations should be considered. PK-DDIs mostly occurred during admission and discharge, indicating that most of the identified clinically significant DDIs could be avoided by simple improvements in prescription practices. Patients were often exposed to PD-DDIs during hospitalization, but under a risk–benefit approach from the treating physicians. Drug categories of CVD (antiarrhythmics, β-blockers, statins, angina), CNS (analgesics, psycholeptics), antithrombotic agents (anticoagulants, antiplatelet), PPIs, and antibiotics often contributed as interacting combinations with other drugs, posing the need for evidence-based evaluations prior to any administration of these drugs. In addition, the study recorded the use of DSs among patients to analyze their daily dietary habits for promoting well-being. The main finding is the regular consumption of grapefruit juice among CTS patients. Although there are numerous data revealing potential conflicts of DS use with medications (such as statins or other CVD drugs), it was also evident that patients did not consider it necessary to inform their treating physicians under the belief that the natural origin of DSs makes them safe to use. The need for improvements regarding prescription practices, continuous vigilance, and constant evaluation for better clinical considerations as well as, communication among patients and healthcare providers is evident from our results. Especially for Greece, healthcare providers and treating physicians should be aware of potential and clinically significant DDIs during hospitalization; additionally, they should often query their patients regarding the potential use of DSs in order for clinically significant drug interactions and related ADRs to be avoided.

## Figures and Tables

**Table 1:** Strengthening the Reporting of Observational Studies in Epidemiology (STROBE) information for the study regarding methods and results.

Methods	
Study design	Analysis of DDIs ^1^ and DDSIs ^2^ in patients admitted to CTS ^3^ clinic in Greece
Setting	Cardiothoracic surgery of University Hospital of Heraklion in Greece
Participants	Patients that need cardiothoracic surgery (CTS) due to progressed CVD ^4^
Variables	Record of demographic characteristics, clinical values, comorbidities, medication regimens, and dietary supplement usage Analyze DDIs and DDSIs, their clinical significance, and pharmacological mechanisms
Data sources/measurement	DDIs, DDSIs and their clinical significance based on literature search and relative databases (Medscape, Drugs.com)
Study size	Target population: patients admitted for CTS Study population: signed informed consent form to participate
Bias	Diligence in informing the purpose and objectives of the study Diligence in recording the medication regimens in correct time periods Recording demographics and medication regiments Analysis of data regarding significance Dietary supplement and self-medication habits feedback
Results	
Participants	76 patients signed informed consent form (95% of total patients in the clinic)
Descriptive data	60.5% male and 39.5 female average age 66 years (min 37, max 85) Average comorbidities: 5 Average hospitalization: 10 days Admittance diagnosis ^5^: CABG (50%), SAVR 24%, CABG/SAVR/MVPL/CEA/BENTALL 16%, AAA 2%
Outcome data	Comorbidities: hypertension, hyperlipidemias and diabetes were most frequent 166 unique DDIs PK-DDIs ^6^: 53 unique combinations PD-DDIs ^7^: 113 unique combinations PK-DDSIs: 18 unique combinations PD-DDSIs: 12 unique combinations
Main results	12% of the identified DDIs were characterized as serious and an alternative should have been used Positive trend between number of medications and prevalence of DDIs regardless the time point PK-DDIs were highly prevalent during admission and discharge whereas PD-DDIs recorded mostly during pre- and postoperation periods. 60% of patients use DS products 60% of the DDSIs were related to PK processes and 36% of the identified PK-DDSIs were due to the consumption of grapefruit juice Patients avoid or neglect seeking medical advice regarding DS usage

^1^ DDIs: drug–drug interactions; ^2^ DDSIs: drug–dietary supplements interactions; ^3^ CTS: cardiothoracic surgery; ^4^ CVD: cardiovascular disease; ^5^ CABG: coronary artery bypass graft surgery; SAVR: surgical aortic valve replacement; MVPl mitral valve prolapse; CEA: carotid endarterectomy; AAA: abdominal aortic aneurysm; Bentall: aortic root replacement surgical procedure; ^6^ PK-DDIs: pharmacokinetic DDIs; ^7^ PD-DDIs: pharmacodynamic DDIs.

**Table 2:** Demographic characteristics, diagnosis, and comorbidities for CVD patients of the study.

**Demographics ^1^**	**Mean (±S.D.)**	**Min/Max**
Age (y)	66 (±10.20)	37/85
Height (m)	1.65 (±0.1)	1.40/1.84
Weight (kg)	81.1 (±15.26)	52.6/122.7
BMI (kg/m^2^)	29.9 (±5.05)	17.1/45.4
Comorbidities	5	1/10
Days hospitalization (d)	10	2/18
Preoperative hospitalization (d)	2	1/8
Postoperative hospitalization (d)	8	2/12
**Diagnosis ^2^**	**Number of Patients (%)**	
CABG	38 (50%)	
SAVR	18 (24%)	
MVP/CEA/Bentall/CABG-SAVR	12(16%)	
AAA	2 (2%)	
Other	6 (8%)	
**Comorbidities**		
Hypertension	64 (84%)	
Diabetes	40 (52%)	
Hyperlipidemias	61 (80%)	
Thyroid	23 (30%)	
Central Nervous System	18 (24%)	
Gastrointestinal	4 (6%)	
Respiratory	5 (4%)	
Other	8 (10%)	
**Social Habits (Smoking & Alcohol)**	**Number of Patients (%)**	
Smoker	25 (32%)	
Ex-smoker	32 (42%)	
Nonsmoker	20 (26%)	
Alcoholic	6 (8%)	
Social drinker	34 (45%)	
Nondrinker	36 (47%)	

^1^ BMI: body mass index; ^2^ diagnosis: CABG: coronary artery bypass graft surgery; SAVR: surgical aortic valve replacement; MVPl mitral valve prolapse; CEA: carotid endarterectomy; AAA: abdominal aortic aneurysm; Bentall: aortic root replacement surgical procedure.

**Table 3:** PK and PD-DDIs identified in the study (see supplementary file for full list and information).

Drug A	Drug B	Drug Categories		Pharmacological Outcome	Number of Cases
**Pharmacokinetic drug interactions: Serious-use alternative**					
amiodarone	acenocoumarol	antiarrhythmic	anticoagulant	CYP * metabolism inhibition acenocoumarol levels	4
amlodipine	simvastatin	Ca^2+^-blocker	antilipidemic	CYP3A4 inhibition (statin-rhabdomyolysis)	4
aspirin	methotrexate	NSAIDs *	rheumatoid arthritis	PK-Renal clearance (methotrexate toxicity)	2
esomeprazole	cilostazol	PPI *	antiplatelet	PK-CYP2C19 inhibition of cilostazol	2
esomeprazole	clopidogrel	PPI	antiplatelet	Reduced antiplatelet activity -CYP2C9 metabolism	11
esomeprazole	escitalopram	PPI	SSRI *	PK-CYP2C19 metabolism inhibition	1
haloperidol	amiodarone	antipsychotic	antiarrhythmic	PK-CYP2D6 inhibition	1
ranolazine	carvedilol	angina	β-blocker	PK-CYP2D6 metabolism (carvedilol)	3
ranolazine	metformin	angina	diabetes II	PK-renal clearance (metformin) OCT2	2
ranolazine	simvastatin	angina	antilipidemic	CYP3A4 inhibition (statin-rhabdomyolysis)	3
**Pharmacokinetic drug interactions: Use with caution-Monitor**					
haloperidol	metoprolol	antipsychotic	β-blocker	PK CYP2D6 metabolism inhibition (metoprolol)	6
atorvastatin	valsartan	antilipidemic	ARBs *	PK-OATB1 * transporter	4
amiodarone	metoprolol	antiarrhythmic	β-blocker	PK-CYP2D6 inhibition for metoprolol (bradycardia)	3
omeprazole	clopidogrel	PPI	antiplatelet	PK CYP2C9 metabolism (clopidogrel)	3
ciprofloxacin	acenocoumarol	antibiotic	anticoagulant	PK-CYP1A2 inhibition acenocoumarol levels	2
**Pharmacokinetic drug interactions: Moderate-Minor**					
budesonide	acenocoumarol	corticosteroid	anticoagulant	PK-CYP3A4 induction metabolism of acenocoumarol	13
amiodarone	codeine	antiarrhythmic	analgesic	PK-CYP2D6 (codeine)	2
carvedilol	haloperidol	β-blocker	antipsychotic	PK-CYP2D6 inhibition	2
ciprofloxacin	alprazolam	antibiotic	anxiolytics	PK CYP3A4 metabolism inhibition (alprazolam)	3
ferrous (gluconate, sulfate etc.)	levothyroxine	anemia	thyroid	PK-T4 GI absorption	4
**Pharmacodynamic drug interactions: Serious-use alternative**					
alprazolam	haloperidol	anxiolytics	antipsychotic	synergism sedation	2
amiloride	potassium chloride	diuretic	hypokalemia	synergism hyperkalemia	3
citalopram	duloxetine	SSRI *	SNRI *	synergism (serotonin syndrome)	1
fenofibrate	pitavastatin	antilipidemic	antilipidemic	synergism	2
haloperidol	amiodarone	antipsychotic	antiarrhythmic	QT prolongation	1
morphine	escitalopram	analgesic	SSRI	serotonin syndrome	1
quetiapine	haloperidol	antipsychotic	antipsychotic	enhance antidopaminergic effect, QT prolongation	6
tramadol	pethidine	analgesic	analgesic	synergism sedation	2
**Pharmacodynamic drug interactions: Use with caution-Monitor**					
alprazolam	morphine	anxiolytics	analgesic	PD-synergism sedation	16
aspirin	acenocoumarol	NSAIDS	antiplatelet	PD-synergism risk of bleeding	11
carvedilol	furosemide	β-blocker	diuretic	PD-antagonism and serum potassium	9
ciprofloxacin	haloperidol	antibiotic	antipsychotic	PD-QT prolongation	3
quetiapine	ipratropium	antipsychotic	anticholinergic	PD-synergism anticholinergic effects, hypoglycemia, QT-prolongation	8
**Pharmacodynamic drug interactions: Moderate-Minor**					
aspirin	perindopril	NSAIDs	ACE *	PD-antagonism kidney (decrease in renal function)	11
bisoprolol	furosemide	β-blocker	diuretic	PD-antagonism (serum potassium)	17
bromazepam	morphine	anxiolytics	analgesic	PD-synergism sedation	25
ceftriaxone	furosemide	antibiotic	diuretic	nephrotoxicity	35
perindopril	enoxaparin	ACE	antiplatelet	PD-hyperkalemia	7

* (SSRI: selective serotonin reuptake inhibitor; SNRI: serotonin-norepinephrine reuptake inhibitor; NSAIDs: nonsteroidal anti-inflammatory drugs; PPI: proton-pump inhibitor; ACE: angiotensin-converting enzyme inhibitor; ARB: angiotensin II receptor blockers; OATB1: organic anion transporter B1).

**Table 4:** DDSIs recorded in the study. Clinical significance (Clin. Sign.): 1-Serious–Use alternative, 2-Use with caution-Monitor, and 3-Moderate-Minor.

Drugs	DS	PK-PD Mechanism	Drug Category	Clin. Sign.	Potential Clinical Outcome	No Cases
metformin	aloe vera	PD	diabetes II	2	hypoglycemia	2
levothyroxine	caffeine	PK-GI absorption	thyroid	2	decreased T4 levels	5
aspirin	fish oil	PD	anticoagulate	2	bleeding	2
clopidogrel	fish oil	PD	anticoagulate	2	bleeding	1
eplerenone	grapefruit	PK-CYP3A4 inhibition	diuretic	2	hyperkaliemia	1
amlodipine	grapefruit	PK-CYP3A4 inhibition	Ca^2+^-blocker	3	mlodipine-ADRs	2
clopidogrel	grapefruit	PK-CYP3A4 inhibition	antiplatelet	2	reduced bioactivation	6
simvastatin	grapefruit	PK-CYP3A4 inhibition	antilipidemic	1	statin-ADRs	1
ranolazine	grapefruit	PK-CYP3A4 inhibition	chronic angina	1	QT prolongation	1
atorvastatin	grapefruit	PK-CYP3A4 inhibition	antilipidemic	1	statin-ADRs	7
tamsulosin	grapefruit	PK-CYP3A4 inhibition	prostatic hyperplasia	2	tamsulosin-ADRs	1
alfuzosin	grapefruit	PK-CYP3A4 inhibition	a1-antagonist-prostate	3	alfuzosin-ADRs	1
finasteride	grapefruit	PK-CYP3A4 inhibition	prostatic hyperplasia	3	finasteride-ADRs	1
alprazolam	grapefruit	PK-CYP3A4 inhibition	anxiety	2	cilostazol-ADRs	1
cilostazol	grapefruit	PK-CYP3A4 inhibition	antiplatelet	2	risk for bleeding	1
ivabradine	grapefruit	PK-CYP3A4 inhibition	angina	1	ivabradine-ADRs	1
ticagrelor	grapefruit	PK-CYP3A4 inhibition	antiplatelet	1	risk for bleeding	1
prasugrel	green tea	PD	antiplatelet	3	Increased drug action	1
aspirin	green tea	PD	antiplatelet	3	bleeding	7
clopidogrel	green tea	PD	antiplatelet	3	bleeding	5
cilostazol	green tea	PD	antiplatelet	3	risk for bleeding	1
ferrous sulfate	green tea	PK-GI absorption	iron deficiency	2	reduced Fe absorption	1
folic acid (FA)	green tea	PK-GI absorption	iron deficiency	2	reduced FA absorption	1
metformin	sage	PD	antidiabetic	3	hypoglycemia	4
alprazolam	sage	PD	anxiety	3	increased sedation	2
gabapentin	sage	PD	anticonvulsant	3	convulsions	1
insulin	sage	PD	diabetes I	3	hypoglycemia	1
sigagliptin	sage	PD	diabetes II	3	hypoglycemia	1
aspirin	B12	PK-GI absorption	B12-deficiency	2	B12-deficiency	1
ensomeprazole	B12	PK-GI absorption	B12-deficiency	3	B12-deficiency	1
					**Total**	**62**

## Data Availability

During the data collection and analysis, all procedures were followed to ensure the confidentiality of the participants in accordance with its EU directives and the General Data Protection Regulation (GDPR). Data presented in the study are available to use on request/ permission from the corresponding author. The data are not publicly available due to privacy statements and ethical reasons that were included in the informed consent form signed by the participants.

## Supplementary Materials

The following are available online at https://www.mdpi.com/1999-4923/13/2/239/s1, Figure S1: Clinical data for patients in the study, Figure S2: Percentages of patients exposed in clinical significant DDIs, Table S1: PK-DDIs- Serious -use alternative, Table S2: PK-DDIs Use with caution -monitor, Table S3: PK-DDIs Moderate-minor, Table S4: PD-DDIs Serious -use alternative, Table S5: PD-DDIs Use with caution -monitor Table S6: PD -DDIs Moderate-minor. Table S7: List of administered drugs and frequency of administration for each time point.
