# Supplementary material for: Prevalence and Clinical Significance of Drug–Drug and Drug–Dietary Supplement Interactions among Patients Admitted for Cardiothoracic Surgery in Greece
Source: Pharmaceutics. 2021 Feb 9;13(2):239. doi: 10.3390/pharmaceutics13020239 (PMC7914879; doi:10.3390/pharmaceutics13020239)
Supplement: Supplementary file 1 [file pharmaceutics-13-00239-s001.pdf]

pharmaceutics

MDPI

Supplementary Materials: Prevalence and Clinical Significance of Drug–Drug and Drug–Dietary Supplement Interactions among Patients Admitted for Cardiothoracic Surgery in Greece

Marios Spanakis, Maria Melissourgaki, George Lazopoulos, Athina E. Patelarou and Evridiki Patelarou

The supplementary file contains additional information regarding the clinical data that were recorded during the study, the percentages of patients exposed in DDIs during hospitalization, the full list of drug interactions that were identified with relative references that support their clinical significance and the list of drugs that were recorded in each time period of hospitalization in the CTS clinic (admission, pre-operation, post-operation, discharge).

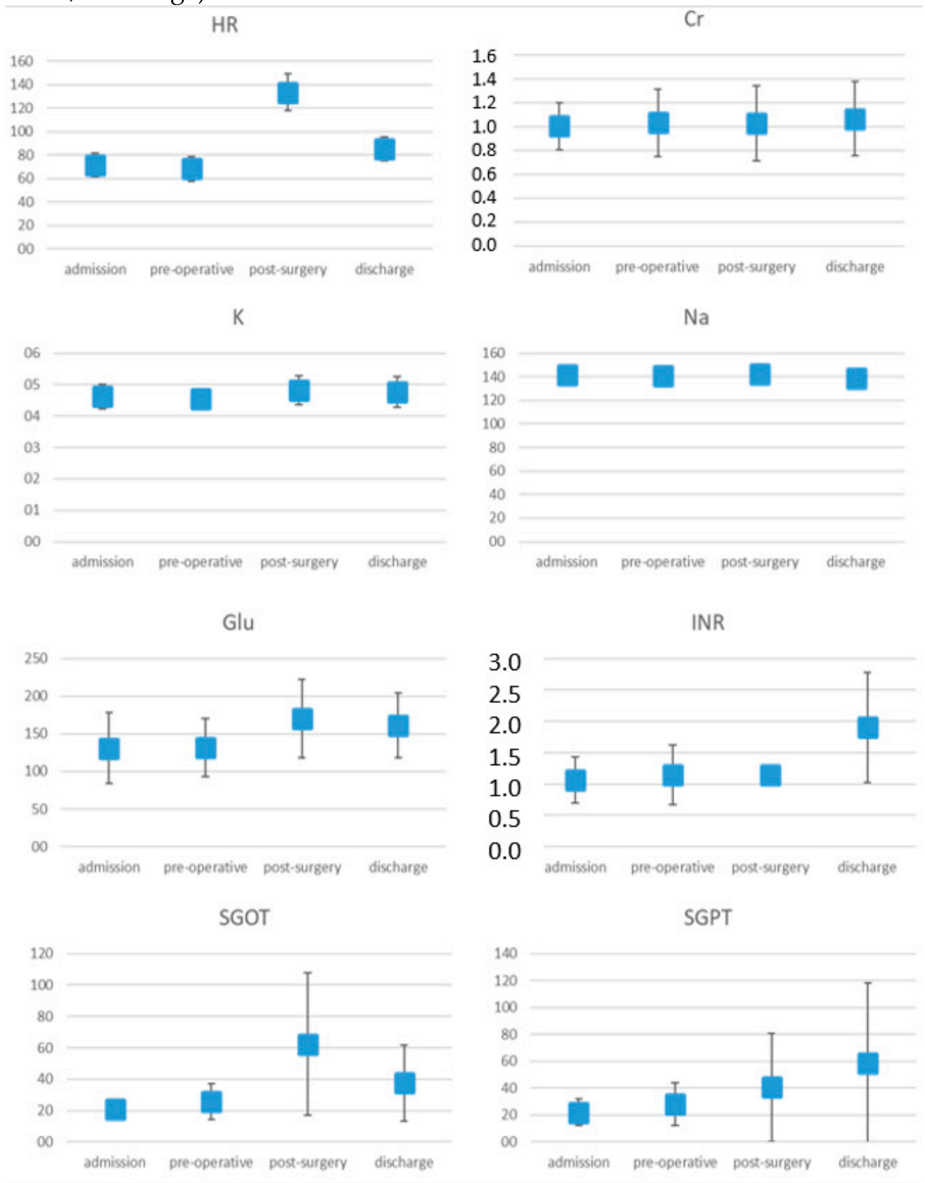

Figure S1. Clinical data for patients in the study (HR: Heart Rate (bmp), Cr: Creatinine (μmol/L), K: Potassium mEq/L, Na: Sodium Chloride mEq/L, INR: International Normalized Ratio, Glu: Blood Glucose mg/dL).

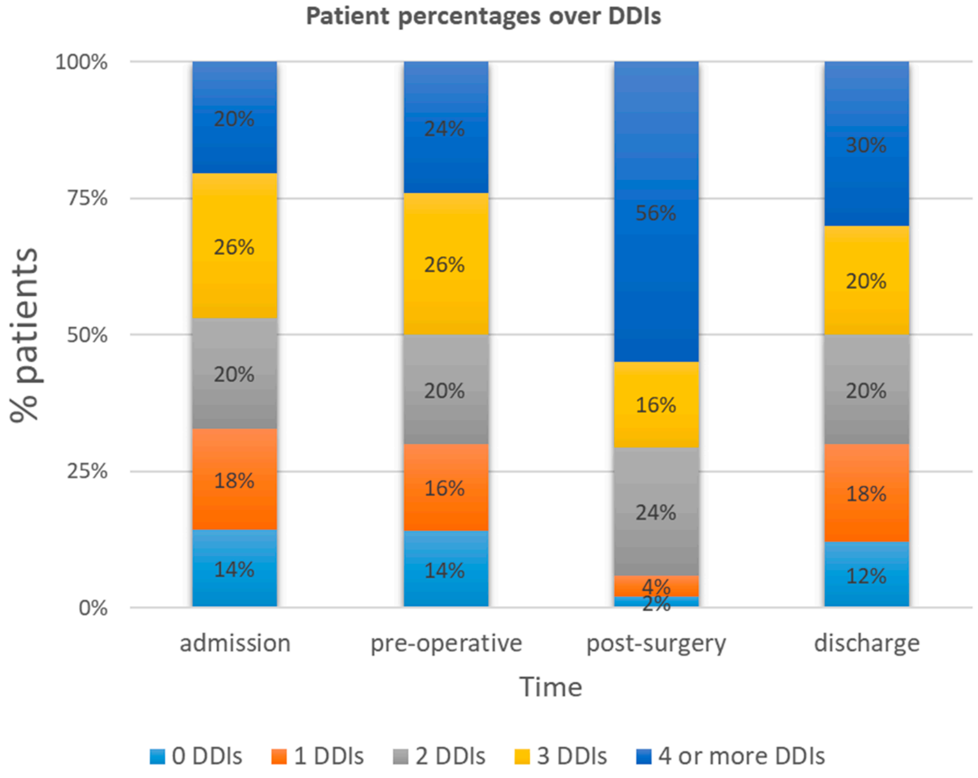

Figure S2. Percentages of patients exposed in clinically significant DDIs.

Table S1. PK- DDIs -Serious-use alternative.

Drug A	Drug B	Drug categories		Pharmacological-Clinical outcome	Number of cases	Medscape	Drugs.com	Refs
**amiodarone**	acenocoumarol	anti-arrhythmic	anticoagulant	PK-CYP metabolism inhibition acenocoumarol levels	4	Serious use alternative	Major	[1]
amlodipine	simvastatin	Ca-blocker	antilipidemic	PK -CYP3A4 inhibition (statin-rhabdomyolisis)	4	Serious use alternative	Major	[2]
aspirin	methotrexate	NSAIDs	chemo-RA	PK-Renal clearance (methotrexate toxicity)	2	Serious use alternative	Major	[3]
esomeprazole	cilostazol	PPI	anti-platelet	PK-CYP2C19 inhibition of cilostazol	2	Serious use alternative	Major	[4]
esomeprazole	clopidogrel	PPI	anti-platelet	reduced antiplatelet activity - CYP2C9 metabolism	11	Serious use alternative	Moderate	[5]
esomeprazole	escitalopram	PPI	SSRI	PK-CYP2C19 metabolsim inhibition	1	Monitor closely	Moderate	[6]
haloperidol	amiodarone	antipsychotic	anti-arrhythmic	PK-CYP2D6 inhibition	1	Serious use alternative	Major	[7]
ranolazine	carvedilol	angina	β-blocker	PK-CYP2D6 metabolism (carvedilol)	3	Monitor closely	Moderate	
ranolazine	metformin	angina	diabetes II	PK -renal clearance (metformin) OCT2	2	Serious use alternative	Moderate	[8]
ranolazine	simvastatin	angina	antilipidemic	CYP3A4 inhibition (statin-rhabdomyolysis)	3	Serious use alternative	Major	[19-20]

Table S2. PK-DDIs Use with caution-Monitor.

Drug A	Drug B	Drug categories		Pharmacological-Clinical outcome	Number of cases	Medscape	Drugs.com	Refs
**allopurinol**	acenocoumarol	uric acid	anticoagulant	PK-CYP1A2 inhibition acenocoumarol levels	1	Serious use alternative	Moderate	[9-11]
amiodarone	atorvastatin	antiarrhythmic	antilipidemic	PK-bioavailability of atorvastatin	2	Monitor closely	Moderate	[12]
amiodarone	metoprolol	antiarrhythmic	β-blocker	PK-CYP2D6 inhibition for metoprolol (bradycardia)	3	Monitor closely	Moderate	[13]
amiodarone	tramadol	antiarrhythmic	analgesic	PK-CYP2D6 inhibition for tramadol	1	Monitor closely	Major	[14]
aspirin	sodium valproate	NSAIDs	antiepileptic	PK-protein binding	2	Monitor closely	Moderate	[15]
atorvastatin	valsartan	antilipidemic	ARBs	PK-OATB1 transporter	4	Monitor closely	Moderate	[16]
carvedilol	amiodarone	α,β-blockers	antiarrhythmic	PK-CYP2D6 inhibition (carvedilol)	1	Monitor closely	Moderate	[13]
ciprofloxacin	acenocoumarol	antibiotic	anticoagulant	PK-CYP1A2 inhibition acenocoumarol levels	2	Serious use alternative	Major	[10,11]
diltiazem	budesonide	angina	corticosteroid	PK-CYP3A4 inhibition for budenoside	1	Serious use alternative	Moderate	[17]
esomeprazole	acenocoumarol	PPI	anticoagulant	PK-CYP2C9 metabolism inhibition	1	Minor	Moderate	[10]
esomeprazole	citalopram	PPI	SSNRI	PK-CYP2C19 inhibition of citalopram	1	Monitor closely	Major	[6]
haloperidol	metoprolol	antipsychotic	β-blocker	PK CYP2D6 metabolism inhibition (metoprolol)	6	Monitor closely	Moderate	[18]
omeprazole	clopidogrel	PPI	anti-platelet	PK CYP2C9 metabolism (clopidogrel)	3	Serious use alternative	Major	[5]
pantoprazole	clopidogrel	PPI	anti-platelet	PK-CYP2C19 inhibition of clopidorgrel	1	Monitor closely	Moderate	[5]
ranolazine	atorvastatin	angina	antilipidemic	PK- myopathy (P-gp and OATB1 inhibition)	2	Monitor closely	Major	[19,20]
ranolazine	rosuvastatin	angina	antilipidemic	PK- myopathy (OATB1 inhibition)	2	Monitor closely	No interaction	[19,20]
ticagrelor	ivabradine	anti-platelet	angina	PK-CYP3A4 inhibition (ivabradine)	1	Serious use alternative	moderate	[21]

Table S3. PK- DDIs Moderate-Minor.

Drug A	Drug B	Drug categories		Pharmacological-Clinical outcome	Number of cases	Medscape	Drugs.com	Refs
acenocoumarol	simvastatin	anticoagulant	antilipidemic	PK-CYP2C9 (polymorphism depedent)	2	Monitor closely	Minor	[10,11]
amiodarone	codeine	antiarrhythmic	analgesic	PK-CYP2D6 (codeine)	2	Monitor closely	Moderate	[14,22,23]
amoxicillin	aspirin	antibiotic	NSAIDs	PK-protein binding /renal clearance	4	Monitor closely	No interaction	[24]
budesonide	acenocoumarol	corticosteroid	anticoagulant	PK-CYP3A4 induction metabolism of acenocoumarol	13	Monitor closely	Moderate	[10,11]
budesonide	alprazolam	corticosteroid	anxiolytics	PK-CYP3A4 induction metabolism	6	Monitor closely	No interaction	[25]
budesonide	amiodarone	corticosteroid	anti-arrhythmic	PK-CYP3A4 induction metabolism	6	Monitor closely	Moderate	[17]
budesonide	clopidogrel	corticosteroid	anti-platelet	PK-CYP3A4 induction metabolism of clopidogrel	5	Monitor closely	No interaction	[17]
budesonide	dutasteride	corticosteroid	BPH	PK-CYP3A4 induction metabolism of dutasteride	1	Minor	No interaction	[17]
budesonide	eplerenone	corticosteroid	diuretic	PK-metabolism induction	5	Minor	Moderate	[17]
budesonide	finasteride	corticosteroid	urologicals	PK-CYP3A4 induction	2	Minor	No interaction	[17]
budesonide	quetiapine	corticosteroid	antipsychotic	PK-CYP3A4 induction	4	Monitor closely	No interaction	[17]
budesonide	ranolazine	corticosteroid	angina	PK-ranolazine	1	Serious use alternative	Moderate	[17]

				inhibits CYP3A4				
carvedilol	haloperidol	β-blocker	antipsychotic	PK-CYP2D6 inhibition	2	Monitor closely	Moderate	[14]
ciprofloxacin	alprazolam	antibiotic	anxiolytics	PK CYP3A4 metabolism inhibition (alprazolam)	3	Minor	No interaction	[14]
domperidone	alprazolam	antiemetic	anxiolytics	reduce domperidone metabolism	10	Not listed	Not listed	[1,2] and [37]
esomeprazole	alprazolam	PPI	anxiolytics	PK CYP3A4 metabolism inhibition (alprazolam)	4	Minor	No interaction	[26]
esomeprazole	cefuroxime	PPI	antibiotic	(both oral forms) modulate of pH -absorption	2	Monitor closely	Moderate	[27]
esomeprazole	duloxetine	PPI	SSRI	PK -duloxetine GI absorption	1	Monitor closely	Minor	[26]
ferrous (gluconate, sulfate)	levothyroxine	anaemia	thyroid	PK -T4 GI absorption	4	Monitor closely	Moderate	[28]
hydrocortisone	acenocoumarol	corticosteroid	anticoagulant	PK-CYP3A4 induction metabolism of acenocoumarol	1	Monitor closely	Moderate	[10]
omeprazole	carvedilol	PPI	β-blocker	PK-CYP2C9 metabolism (carvedilol)	2	Minor	Moderate	[26]
ranitidine	metformin	H2-receptor antagonist	diabetes II	PK- renal clearance (metform)	2	No interaction	Moderate	[29]
ranitidine	vitamin B12	H2-receptor antagonist	vitamin B12	PK-GI absorption for B12	15	No interaction	Minor	[30]
ranolazine	metoprolol	angina	β-blocker	PK-CYP2D6 metabolism (metoprolol)	1	Monitor closely	Moderate	[31]

[1] https://www.mayoclinic.org/drugs-supplements/alprazolam-oral-route/precautions/drg-20061040?p=1
[2] https://www.medicines.org.uk/emc/product/556/smpc

ranolazine	morphine	angina	analgesic	PK-inhibition CYP2D6 increase morphine levels	1	Monitor closely	Moderate	[31]
tamsulosin	haloperidol	BPH	antipsychotic	PK-CYP2D6 inhibition	1	Monitor closely	Moderate	[32]

Table S4. PD- DDIs Serious -use alternative.

Drug A	Drug B	Drug categories		Pharmacological-Clinical outcome	Number of cases	Medscape	Drugs.com	Refs
alprazolam	haloperidol	anxiolytics	antipsychotic	PD-synergism sedation	2	Monitor closely	Moderate	[33]
amiloride	potassium chloride	diuretic	hypokalemia	PD-synergism hyperkalemia	3	Serious use alternative	Major	[34]
citalopram	duloxetine	SSNRI	SSRI	PD-synergism (Serotonine syndrome)	1	Serious use alternative	Major	[35]
fenofibrate	pitavastatin	antilipidemic	antilipidemic	PD-synergism	2	Serious use alternative	Major	[36]
haloperidol	amiodarone	antipsychotic	anti-arrhythmic	PD-QT prolangation	1	Serious use alternative	Major	[37]
morphine	escitalopram	analgesic	SSRI	PD-serotonin syndrome	1	Serious use alternative	Moderate	[38]
quetiapine	haloperidol	antipsychotic	antipsychotic	PD-enhance antidopaminergic effect -QT prolangation	6	Monitor closely	Major	[33]
tramadol	pethidine	analgesic	analgesic	PD-synergism sedation	2	Serious use alternative	Major	

Table S5. PD-DDIs Use with caution -monitor.

Drug A	Drug B	Drug categories		Pharmacological-Clinical outcome	Number of cases	Medscape	Drugs.com	Refs
alprazolam	morphine	anxiolytics	analgesic	PD-synergism sedation	16	Monitor closely	Major	[39]
alprazolam	quetiapine	anxiolytics	antipsychotic	PD-synergism sedation	4	Monitor closely	Major	
amlodipine	metformin	Ca-blocker	diabetes II	PD-antagonism (metformin action)	4	Monitor closely	No interaction	[40]
apixaban	aspirin	anticoagulant	NSAIDs	PD-synergism	1	Monitor closely	Major	[41]
aspirin	acenocoumarol	NSAIDS	anti-platelet	PD synergism	11	Monitor closely	Major	[10]
aspirin	carvedilol	NSAIDs	β-blocker	PD antagonism and serum potassium	11	Monitor closely	Minor	[42]

aspirin	citalopram	NSAIDs	SSNRI	PD-synergism (bleeding)	1	Monitor closely	Moderate	[43]
aspirin	duloxetine	NSAIDs	SSRI	PD-synergism (bleeding)	1	Monitor closely	Moderate	[43]
aspirin	escitalopram	NSAIDs	SSRI	PD-synergism (bleeding)	1	Monitor closely	Moderate	[43]
aspirin	rivaroxaban	NSAIDs	anticoagulant	PD-synergism	1	Monitor closely	Major	[41]
bisoprolol	norepinephrine	β-blocker	adrenergic	PD-antagonism	2	Monitor closely	Moderate	[44]
bromazepam	haloperidol	anxiolytics	antipsychotic	PD-synergism sedation	2	Monitor closely	Moderate	[33]
bromazepam	olanzapine	anxiolytics	antipsychotic	PD-synergism sedation	1	Monitor closely	Major	[45]
bromazepam	quetiapine	anxiolytics	antipsychotic	PD-synergism sedation	1	Monitor closely	Moderate	[46]
carvediylol	amlodipine	β-blocker	Ca-blocker	PD- synergism	4	Monitor closely	Moderate	[47]
carvedilol	furosemide	β-blocker	diuretic	PD antagonism and serum potassium	9	Monitor closely	Moderate	[47]
carvedilol	hydrochlorothiazide	β-blocker	diuretic	PD-antagonism (serum pottasium)	1	Monitor closely	Moderate	
carvedilol	irbesartan	β-blocker	ARBs	PD-synergism (serum pottasium)	4	Monitor closely	No interaction	
ceftriaxone	acenocoumarol	antibiotic	anticoagulant	PD -risk of bleeding	13	Monitor closely	Moderate	[10]
ceftriaxone	enoxaparin	antibiotic	anti-platelet	increases effects of enoxaparin	21	Serious use alternative	No interaction	[48]
ciprofloxacin	amiodarone	antibiotic	antiarrhythmic	PD-QT prolongation	1	Monitor closely	Major	[49]
ciprofloxacin	haloperidol	antibiotic	antipsychotic	PD -QT prolongation	3	Monitor closely	Major	[37]
clopidogrel	acenocoumarol	anti-platelet	anticoagulant	PD-synergism	1	Monitor closely	Major	[10]
clopidogrel	citalopram	anti-platelet	SSNRI	PD-synergism (bleeding)	1	Monitor closely	Moderate	[43]
clopidogrel	duloxetine	anti-platelet	SSRI	PD-synergism (bleeding)	1	Monitor closely	Moderate	[43]
codeine	tramadol	analgesic	analgesic	PD-synergism sedation	1	Monitor closely	Major	
domperidone	hydroxyzine	antiemetic	anxiolytics	PD-QT prolongation	2	Not listed	Not listed	[50]
enoxaparin	acenocoumarol	anti-platelet	anticoagulant	PD-synergism	8	Serious use alternative	Major	[10]
fenofibrate	acenocoumarol	antilipidemic	anticoagulant	PD-synergism	1	Serious Use alternative	Major	[10]

furosemide	empagli-flozin	diuretic	diabetes II	PD-synergism	2	Monitor closely	Moderate	[51]
haloperidol	ipratropium	antipsychotic	anticholiner-gic	PD -synergism additive anti-cholinergic ef-fects, hypogly-cemia	7	Monitor closely	Moderate	[33]
haloperidol	lorazepam	antipsychotic	anxiolytics	PD-synergism sedation	1	Monitor closely	Moderate	[33]
haloperidol	melatonin	antipsychotic	sedative	PD-synergism sedation	2	Monitor closely	No interac-tions	[33]
haloperidol	morphine	antipsychotic	analgesic	PD-synergism sedation	1	Monitor closely	Major	[33]
haloperidol	tiotropium	antipsychotic	anticholiner-gic	PD -synergism additive anti-cholinergic ef-fects, hypogly-cemia	1	Monitor closely	Moderate	[33]
hydrocorti-sone	enoxaparin	corticosteroid	anti-platelet	reduced anti-platelet activity	1	Monitor closely	No interac-tions	[52]
hydroxyzine	morphine	anxiolytics	analgesic	PD synergism	3	Monitor closely	Major	[39]
lisinopril	aspirin	ACE	NSAIDs	PD -antago-nism (decrease renal function)	2	Serious use alternative	Moderate	[47]
methylpred-nisolone	enoxaparin	corticosteroid	anti-platelet	reduced anti-platelet activity	1	Monitor closely	No interac-tions	[52]
metoprolol	furosemide	β-blocker	diuretic	PD -antago-nism (serum pottasium)	25	Monitor closely	Moderate	[47]
metoprolol	norepineph-rine	β-blocker	adrenergic	PD-antago-nism -potential diminished cardiac re-sponse	4	Monitor closely	Moderate	[53]
olanzapine	ipratropium	antipsychotic	anticholiner-gic	PD -synergism additive anti-cholinergic ef-fects, hypogly-cemia	2	Monitor closely	Moderate	[54]
olanzapine	metformin	antipsychotic	diabetes II	PD-synergism hypoglycemia	2	Monitor closely	Moderate	[54]
olanzapine	morphine	antipsychotic	analgesic	PD-synergism sedation	1	Monitor closely	Major	[54]
olanzapine	pethidine	antipsychotic	analgesic	PD-serotonin syndrome	1	Monitor closely	Major	[54]
olanzapine	sitagliptin	antipsychotic	diabetes II	PD-synergism hypoglycemia	2	Monitor closely	Moderate	[54]
olmesartan	metformin	ARBs	diabetes II	PD-synergism (lactic acidosis)	1	No interac-tion	No interac-tion	[55]

perindopril	metformin	ACE	diabetes II	PD-synergism (hypoglycemia)	6	Monitor closely	Moderate	[55]
piperacillin	acenocoumarol	antibiotic	anticoagulant	PD-synergism anticoagulation	2	Serious use alternative	Moderate	[10,11]
quetiapine	ipratropium	antipsychotic	anticholinergic	PD -synergism anticholinergic effects, hypoglycemia, QT-prol	8	Monitor closely	Moderate	[56]
quetiapine	morphine	antipsychotic	analgesic	PD-synergism sedation	2	Monitor closely	Major	[39]
quetiapine	tiotropium	antipsychotic	anticholinergic	PD -synergism anticholinergic effects, hypoglycemia, QT-prol	1	Monitor closely	Moderate	[56]
zopiclone	bromazepam	hypnotic	anxiolytics	PD-synergism	1	Monitor closely	Moderate	[57]
zopiclone	morphine	hypnotic	analgesic	PD-synergism	1	No interaction	Major	[57]

Table S6. PD-DDIs moderate-minor.

Drug A	Drug B	Drug categories		Pharmacological-Clinical outcome	Number of cases	Medscape	Drugs.com	Refs
aspirin	furosemide	NSAIDs	diuretic	PD antagonism and serum potassium	44	Monitor closely-Minor	Minor	[47]
aspirin	gliclazide	NSAIDs	diabetes II	PD -hypoglycemia	3	Not listed	Not listed	[58]
aspirin	glimepiride	NSAIDs	diabetes II	PD -hypoglycemia	2	Monitor closely-Minor	Moderate	[58]
aspirin	insulin	NSAIDs	diabetes I	PD synergism	22	Monitor closely	Moderate	[58]
aspirin	irbesartan	NSAIDs	ARBs	PD-antagonism (kidney function)	6	Monitor closely-Minor	Moderate	[59]
aspirin	perindopril	NSAIDs	ACE	PD -antagonism kidney (decrease in renal function)	11	Serious use alternative	Moderate	[47]
aspirin	ramipril	NSAIDs	ACE	PD -antagonism kidney (decrease in renal function)	6	Serious use alternative	Moderate	[47]
aspirin	valsartan	NSAIDs	ARBs	PD-antagonism (kidney function)	3	Monitor closely	Moderate	[59]
biperiden	alprazolam	parkinson	anxiolytics	PD-synergism CNS and Respiration depression	4	Not listed	Moderate	[60]
biperiden	haloperidol	parkinson	antipsychotic	PD-synergism anticholinergic effect	2	Not listed	Moderate	[60]

biperi-den	ipratropium	parkinson	anticholiner-gic	PD-synergism an-ticholinergic effect	2	Not listed	Moderate	[60]
biperi-den	quetiapine	parkinson	antipsychotic	PD-synergism an-ticholinergic effect	4	Not listed	Moderate	[60]
biperi-den	tiotropium	parkinson	anticholiner-gic	PD-synergism an-ticholinergic effect	1	Not listed	Moderate	[60]
biperi-den	valproic acid	parkinson	anticonvul-sant	PD-synergism CNS and Respira-tion depression	1	Not listed	Moderate	[60]
bi-sopro-lol	dobutamine	β-blocker	β-1-agonist	PD -antagonism (serum pottasium)	1	Monitor closely	Moderate	[61]
bi-sopro-lol	formoterol	β-blocker	β-2-agonist	PD-antagonism (b2-receptors)	2	Monitor closely	Moderate	[62]
bi-sopro-lol	furosemide	β-blocker	diuretic	PD-antagonism (serum potassium) (pt2)	17	Monitor closely	Moderate	[47]
brom-azepam	morphine	anxiolytics	analgesic	PD-synergism se-dation	25	Monitor closely	Major	[39]
ceftri-axone	furosemide	antibiotic	diuretic	nehprotoxicity	35	Minor	Moderate	[63]
ciprof-loxacin	insulin	antibiotic	diabetes I	PD synergism hy-poglycaemia	2	Monitor closely	Major	[63]
codeine	morphine	analgesic	analgesic	PD -synergism	3	Monitor closely	Major	[22,39]
codeine	pethidine	analgesic	analgesic	PD-synergism se-dation	17	Monitor closely	Major	[22,39]
dobuta-mine	agomelatine	β-1-agonist	antidepres-sant	PD-antagonism	1	Not listed	Not listed	
dobuta-mine	carvedilol	β-1-agonist	α,β-blockers	PD-antagonism (serum pottasium)	1	Monitor closely	moderate	
domper idone	alfuzosin	antiemetic	BPH	PD synergism - QTc prolongation	3	Not listed	Not listed	[37]
domper idone	amlodipine	antiemetic	Ca-blocker	PD synergism - QTc prolongation	5	Not listed	Not listed	[37]
domper idone	haloperidol	antiemetic	antipsychotic	PD-QT prolanga-tion	1	Not listed	Not listed	[33]
em-pagli-flozin	amiloride	diabetes II	diuretic	PD-synergism (in-creased urine vol-ume)	1	Monitor closely	Moderate	
em-pagli-flozin	furosemide	diabetes II	diuretic	PD-synergism (in-creased urine vol-ume)	2	Monitor closely	Moderate	
enoxap arin	irbesartan	anti-platelet	ARBs	PD- hyperkalemia	1	Monitor closely	Moderate	[64]
enoxap arin	ramipril	anti-platelet	ACE	PD-suppress al-dosterone secre-tion-hyperkalemia	7	Monitor closely	Moderate	[64]
enoxap arin	valsartan	anti-platelet	ARBs	PD- hyperkalemia	1	Monitor closely	Moderate	[64]

hy-droxyz-ine	alprazolam	anxiolytics	anxiolytics	PD -synergism increase sedation	1	Monitor closely	No interaction	[65]
hy-droxyz-ine	bromazepam	anxiolytics	anxiolytics	PD -synergism increase sedation	1	Monitor closely	No interaction	[65]
irbesar-tan	insulin	ARBs	diabetes I	PD-ARBs enhance hypoglycemia	1	Monitor closely	Moderate	[66]
lis-inopril	enoxaparin	ACE	anti-platelet	PD-hyperkalemia	2	Monitor closely	Moderate	[64]
lis-inopril	insulin	ACE	diabetes I	PD-synergism	5	Monitor closely	Moderate	[67]
metopr olol	dobutamine	β-blocker	β-1-agonist	PD -antagonism (serum pottasium)	2	Monitor closely	Moderate	
metopr olol	formoterol	β-blocker	β-2-agonist	PD-antagonism (b2-receptors)	1	Monitor closely	Moderate	
mor-phine	melatonin	analgesic	sedative	PD-synergism sedation	1	Monitor closely	No interaction	[68]
mor-phine	pregabalin	analgesic	antiepileptic	PD-synergism sedation	1	Monitor closely	Major	
olmesar tan	enoxaparin	ARBs	anti-platelet	PD-hyperkalemia	2	Monitor closely	Moderate	
olmesar tan	insulin	ARBs	diabetes I	PD -hypoglycemic effect	5	Monitor closely	Moderate	[66]
perin-dopril	enoxaparin	ACE	anti-platelet	PD-hyperkalemia	7	Monitor closely	Moderate	[64]
perin-dopril	furosemide	ACE	diuretic	PD-synergism	2	Monitor closely	Moderate	[47]
perin-dopril	insulin	ACE	diabetes I	PD-synergism	5	Monitor closely	Moderate	[67]
perin-dopril	pregabalin	ACE	antiepileptic	angioedema	3	Serious use alternative	Moderate	[67]
queti-apine	insulin	antipsychotic	diabetes I	PD-hyperglycemia	3	Monitor closely	Moderate	[69]
queti-apine	metformin	antipsychotic	diabetes II	PD-antagonism glucose levels	6	Monitor closely	Moderate	[69]
rami-pril	furosemide	ACE	diuretic	PD-synergism	9	Monitor closely	Moderate	[47]
valsar-tan	insulin	ARBs	diabetes I	PD-ARBs hypoglycemic effect	2	Monitor closely	Moderate	[66]

Table S7. List of administered drugs and frequency of administration for each time point.

	Admission	Pre-operation	Post-operation	Discharge
acenocoumarol			38%	**38%**
acetylcysteine	2%	**58%**	**86%**	6%
agomelatine	2%	2%	2%	2%
alendronic acid	2%			2%
alfacalcidol	2%			2%
alfuzosin	6%	6%	6%	6%
allopurinol	10%	10%	10%	10%

alogliptin(+metformin)	6%			6%
alprazolam	12%	28%	4%	4%
amiloride			6%	4%
amiodarone			18%	8%
amlodipine	14%	18%		12%
amoxicillin (+/- clavulanic acid)	2%			2%
apixaban	2%			2%
aspirin	**68%**	2%	**60%**	**68%**
atorvastatin (+/- ezetimide)	**60%**	**34%**	4%	**16%**
biperiden HCl	2%	2%		2%
bisacodyl	6%			
bisoprolol	**32%**	**30%**	20%	**20%**
bromazepam	2%	**56%**	6%	2%
budesonide (+/- formoterol)	6%	**86%**	**94%**	2%
calcium/cholecalciferol	2%			2%
carvedilol	18%	16%	8%	12%
ceftriaxone		14%	**82%**	
cefuroxime		2%		2%
cilostazol	2%	2%		2%
ciprofloxacin		8%	6%	2%
citalopram	2%			
clopidogrel	**36%**		8%	12%
codeine			2%	
dapagliflozin	2%		2%	
diltiazem	2%	2%		
dobutamine			30%	
domperidone	2%	**86%**		2%
duloxetine	2%			
empagliflozin	4%			6%
enoxaparin		**50%**	50%	
eplerenone	6%	6%	4%	4%
escitalopram	2%	2%		
esomeprazole	**30%**	12%	16%	8%
ezetimibe	8%	8%		8%
fenofibrate	6%	2%	2%	6%
ferrous (all salts +/- foli acid)	6%			4%
finasteride	2%	2%	2%	2%
fluticasone/salmeterol	2%			
folic acid	4%	2%		4%
fondaparinux		2%		
furosemide	16%	12%	**100%**	**64%**
furosemide (+amiloride)	2%		**82%**	10%
gabapentin	2%			
gliclazide	4%			4%
glimepiride	2%			2%

glycopyrronium bromide	2%			
haloperidol	4%	2%	12%	10%
human serum albumin			2%	
hydrocortisone		2%	2%	
hydroxychloroquine	4%	2%	2%	4%
hydroxyzine		6%		
indapamide	2%			2%
Insulin (all sub-types)	**22%**	10%	38%	**22%**
ipratropium bromide		**88%**	**100%**	
irbesartan (+/- hydrochlorothiazide)	4%	10%	2%	4%
isosorbide mononitrate	16%	10%		
ivabradine	4%	6%	4%	10%
lactulose		2%		
lansoprazole	16%	2%		
lercanidipine	10%	2%		
letrozole	4%			
levothyroxine	18%	14%	18%	**18%**
linezolid				2%
lisinopril	2%	4%	2%	2%
lorazepam			2%	
magnesium aspartate HCl	10%	20%		
melatonin	4%	2%		2%
metformin (+/- empagliflozin/stiagliptin/vildagliptin)	**26%**			**26%**
methotrexate	2%			2%
methylprednisolone		4%		
metoprolol	**20%**	16%	28%	**46%**
morphine		**82%**		
moxonidine	4%	2%		
nebivolol	2%	2%		
nifedipine		2%		
nitroglycerin	4%	4%	10%	
norepinephrine			18%	
olanzapine	2%	2%	2%	2%
olmesartan (+/- medoxomil/amlodipine/hydrochlorothiazide)	8%	8%	2%	6%
omeprazole	8%	4%		
ondansetron			2%	
paracetamol (+/- codeine)	8%		**84%**	
perindopril (+/- amlodipine or indamamide/amlodipine).	**20%**	18%	6%	4%
pethidine			40%	

piperacillin (+/- tazobactam)		2%	2%	
piperacillin		4%	12%	
pitavastatin				
pitavastatin	2%	2%		2%
potassium chloride			8%	
prasugrel	2%			
pregabalin	4%	4%	2%	4%
propafenone				2%
quetiapine	6%	4%	4%	6%
ramipril (+/- hydrochlorothiazide)	**24%**	16%	4%	6%
ranitidine	18%	**80%**	**84%**	**84%**
ranolazine	8%	2%		
rivaroxaban	2%			2%
rosuvastatin	10%	10%		10%
simvastatin (+/- ezetimibe)	12%	6%	2%	8%
sitagliptin (+/- metformin)	4%			4%
Sodium valproate	2%		2%	2%
spironolactone	2%	2%		
tamsulosin (HCL +/- dutasteride)	6%	6%	6%	6%
telmisartan/amlodipine	2%	2%		
ticagrelor	8%			
tiotropium bromide	6%			
tramadol			8%	
trimetazidine	2%	2%		2%
valsartan (+/- amlodipine or hydrochlorothiazide or amoldipine/hydrochlorothiazide)	18%	10%		
vancomycin		2%	**72%**	
vildagliptin (+/- metformin)	6%			8%
Vitamin B1,6,12				2%
zofenopril	2%	2%		
zopiclone	2%	2%	2%	2%

References

1. Caraco Y, Chajek-Shaul T (1989) The incidence and clinical significance of amiodarone and acenocoumarol interaction. Thrombosis and haemostasis 62 (3):906-908
2. Nishio S, Watanabe H, Kosuge K, Uchida S, Hayashi H, Ohashi K (2005) Interaction between amlodipine and simvastatin in patients with hypercholesterolemia and hypertension. Hypertension research : official journal of the Japanese Society of Hypertension 28 (3):223-227. doi:10.1291/hypres.28.223
3. Colebatch AN, Marks JL, Edwards CJ (2011) Safety of non-steroidal anti-inflammatory drugs, including aspirin and paracetamol (acetaminophen) in people receiving methotrexate for inflammatory arthritis (rheumatoid arthritis, ankylosing spondylitis, psoriatic arthritis, other spondyloarthritis). The Cochrane database of systematic reviews (11):CD008872. doi:10.1002/14651858.CD008872.pub2
4. Suri A, Bramer SL (1999) Effect of omeprazole on the metabolism of cilostazol. Clinical pharmacokinetics 37 Suppl 2:53-59. doi:10.2165/00003088-199937002-00006

5. Chua D, Bolt J, Lo A, Lo A (2010) Clopidogrel and proton pump inhibitors: a new drug interaction? The Canadian journal of hospital pharmacy 63 (1):47-50. doi:10.4212/cjhp.v63i1.869
6. Gjestad C, Westin AA, Skogvoll E, Spigset O (2015) Effect of proton pump inhibitors on the serum concentrations of the selective serotonin reuptake inhibitors citalopram, escitalopram, and sertraline. Therapeutic drug monitoring 37 (1):90-97. doi:10.1097/FTD.0000000000000101
7. Bush SE, Hatton RC, Winterstein AG, Thomson MR, Woo GW (2008) Effects of concomitant amiodarone and haloperidol on Q-Tc interval prolongation. American journal of health-system pharmacy : AJHP : official journal of the American Society of Health-System Pharmacists 65 (23):2232-2236. doi:10.2146/ajhp080039
8. Zack J, Berg J, Juan A, Pannacciulli N, Allard M, Gottwald M, Zhang H, Shao Y, Ben-Yehuda O, Jochelson P (2015) Pharmacokinetic drug-drug interaction study of ranolazine and metformin in subjects with type 2 diabetes mellitus. Clinical pharmacology in drug development 4 (2):121-129. doi:10.1002/cpdd.174
9. Gavronski M, Hartikainen S, Zharkovsky A (2012) Analysis of potential interactions between warfarin and prescriptions in Estonian outpatients aged 50 years or more. Pharmacy practice 10 (1):9-16
10. Gschwind L, Rollason V, Lovis C, Boehlen F, Bonnabry P, Dayer P, Desmeules JA (2013) Identification and weighting of the most critical "real-life" drug-drug interactions with acenocoumarol in a tertiary care hospital. European journal of clinical pharmacology 69 (3):617-627. doi:10.1007/s00228-012-1358-7
11. Rojo Sanchis A, Parro Martin M, Delgado Silveira E, Álvarez Diaz A, Pérez Menéndez-Conde C, Bermejo Vicedo T (2014) DI-084 Acenocoumarol drug interactions in hospitalised patients. European Journal of Hospital Pharmacy: Science and Practice 21 (Suppl 1):A104-A104. doi:10.1136/ejhpharm-2013-000436.255
12. Borders-Hemphill V (2009) Concurrent use of statins and amiodarone. The Consultant pharmacist : the journal of the American Society of Consultant Pharmacists 24 (5):372-379. doi:10.4140/tcp.n.2009.372
13. Boutitie F, Boissel JP, Connolly SJ, Camm AJ, Cairns JA, Julian DG, Gent M, Janse MJ, Dorian P, Frangin G (1999) Amiodarone interaction with beta-blockers: analysis of the merged EMIAT (European Myocardial Infarct Amiodarone Trial) and CAMIAT (Canadian Amiodarone Myocardial Infarction Trial) databases. The EMIAT and CAMIAT Investigators. Circulation 99 (17):2268-2275. doi:10.1161/01.cir.99.17.2268
14. Lynch T, Price A (2007) The effect of cytochrome P450 metabolism on drug response, interactions, and adverse effects. American family physician 76 (3):391-396
15. Sandson NB, Marcucci C, Bourke DL, Smith-Lamacchia R (2006) An interaction between aspirin and valproate: the relevance of plasma protein displacement drug-drug interactions. The American journal of psychiatry 163 (11):1891-1896. doi:10.1176/ajp.2006.163.11.1891
16. Kalliokoski A, Niemi M (2009) Impact of OATP transporters on pharmacokinetics. British journal of pharmacology 158 (3):693-705. doi:10.1111/j.1476-5381.2009.00430.x
17. Daveluy A, Raignoux C, Miremont-Salame G, Girodet PO, Moore N, Haramburu F, Molimard M (2009) Drug interactions between inhaled corticosteroids and enzymatic inhibitors. European journal of clinical pharmacology 65 (7):743-745. doi:10.1007/s00228-009-0653-4
18. Shin JG, Kane K, Flockhart DA (2001) Potent inhibition of CYP2D6 by haloperidol metabolites: stereoselective inhibition by reduced haloperidol. British journal of clinical pharmacology 51 (1):45-52. doi:10.1046/j.1365-2125.2001.01313.x
19. Correa D, Landau M (2013) Ranolazine-induced myopathy in a patient on chronic statin therapy. Journal of clinical neuromuscular disease 14 (3):114-116. doi:10.1097/CND.0b013e31828525a5
20. Worz CR, Bottorff M (2001) The role of cytochrome P450-mediated drug-drug interactions in determining the safety of statins. Expert opinion on pharmacotherapy 2 (7):1119-1127. doi:10.1517/14656566.2.7.1119
21. Di Serafino L, Rotolo FL, Boggi A, Colantonio R, Serdoz R, Monti F (2014) Potential additive effects of ticagrelor, ivabradine, and carvedilol on sinus node. Case reports in cardiology 2014:932595. doi:10.1155/2014/932595
22. Caraco Y, Sheller J, Wood AJ (1996) Pharmacogenetic determination of the effects of codeine and prediction of drug interactions. The Journal of pharmacology and experimental therapeutics 278 (3):1165-1174
23. Zhou SF (2009) Polymorphism of human cytochrome P450 2D6 and its clinical significance: part II. Clinical pharmacokinetics 48 (12):761-804. doi:10.2165/11318070-000000000-00000
24. Zhang J, Sun Y, Wang R, Zhang J (2019) Gut Microbiota-Mediated Drug-Drug Interaction between Amoxicillin and Aspirin. Scientific reports 9 (1):16194. doi:10.1038/s41598-019-52632-5
25. Pirmohamed M (2004) The top 100 drug interactions. A Guide to Patient Management. 2003 Edition. Postgraduate Medical Journal 80 (940):123-123
26. Ogawa R, Echizen H (2010) Drug-drug interaction profiles of proton pump inhibitors. Clinical pharmacokinetics 49 (8):509-533. doi:10.2165/11531320-000000000-00000
27. Hughes GS, Heald DL, Barker KB, Patel RK, Spillers CR, Watts KC, Batts DH, Euler AR (1989) The effects of gastric pH and food on the pharmacokinetics of a new oral cephalosporin, cefpodoxime proxetil. Clinical pharmacology and therapeutics 46 (6):674-685. doi:10.1038/clpt.1989.204
28. Campbell NR, Hasinoff BB, Stalts H, Rao B, Wong NC (1992) Ferrous sulfate reduces thyroxine efficacy in patients with hypothyroidism. Annals of internal medicine 117 (12):1010-1013. doi:10.7326/0003-4819-117-12-1010
29. Pakkir Maideen NM, Jumale A, Balasubramaniam R (2017) Drug Interactions of Metformin Involving Drug Transporter Proteins. Advanced pharmaceutical bulletin 7 (4):501-505. doi:10.15171/apb.2017.062

30. Salom IL, Silvis SE, Doscherholmen A (1982) Effect of cimetidine on the absorption of vitamin B12. Scandinavian journal of gastroenterology 17 (1):129-131. doi:10.3109/00365528209181056
31. EMA (2013) Ranexa, Summary of product characteristics. EMA, EMA
32. Andes D, Azie N, Yang H, Harrington R, Kelley C, Tan RD, Wu EQ, Franks B, Kristy R, Lee E, Khandelwal N, Spalding J (2016) Drug-Drug Interaction Associated with Mold-Active Triazoles among Hospitalized Patients. Antimicrobial agents and chemotherapy 60 (6):3398-3406. doi:10.1128/AAC.00054-16
33. EMA (2007) Aloperidin, Summary of Product Characteristics.
34. Perazella MA (2000) Drug-induced hyperkalemia: old culprits and new offenders. The American journal of medicine 109 (4):307-314. doi:10.1016/s0002-9343(00)00496-4
35. Tintinalli JE, Stapczynski JS, Ma OJ, Yealy DM, Meckler GD, Cline DM Tintinalli's emergency medicine: a comprehensive study guide. 9th. edn.,
36. Farnier M (2003) Combination therapy with an HMG-CoA reductase inhibitor and a fibric acid derivative: a critical review of potential benefits and drawbacks. American journal of cardiovascular drugs : drugs, devices, and other interventions 3 (3):169-178. doi:10.2165/00129784-200303030-00003
37. Armahizer MJ, Seybert AL, Smithburger PL, Kane-Gill SL (2013) Drug-drug interactions contributing to QT prolongation in cardiac intensive care units. Journal of critical care 28 (3):243-249. doi:10.1016/j.jcrc.2012.10.014
38. Sproule BA, Naranjo CA, Brenmer KE, Hassan PC (1997) Selective serotonin reuptake inhibitors and CNS drug interactions. A critical review of the evidence. Clinical pharmacokinetics 33 (6):454-471. doi:10.2165/00003088-199733060-00004
39. FDA (2016) FDA Drug Safety Communication: FDA warns about serious risks and death when combining opioid pain or cough medicines with benzodiazepines; requires its strongest warning.
40. Zaman Huri H, Fun Wee H (2013) Drug related problems in type 2 diabetes patients with hypertension: a cross-sectional retrospective study. BMC endocrine disorders 13:2. doi:10.1186/1472-6823-13-2
41. Oldgren J, Wallentin L, Alexander JH, James S, Jonelid B, Steg G, Sundstrom J (2013) New oral anticoagulants in addition to single or dual antiplatelet therapy after an acute coronary syndrome: a systematic review and meta-analysis. European heart journal 34 (22):1670-1680. doi:10.1093/eurheartj/eht049
42. Lindenfeld J, Robertson AD, Lowes BD, Bristow MR, Investigators M (2001) Aspirin impairs reverse myocardial remodeling in patients with heart failure treated with beta-blockers. Journal of the American College of Cardiology 38 (7):1950-1956. doi:10.1016/s0735-1097(01)01641-2
43. Andrade C, Sandarsh S, Chethan KB, Nagesh KS (2010) Serotonin reuptake inhibitor antidepressants and abnormal bleeding: a review for clinicians and a reconsideration of mechanisms. The Journal of clinical psychiatry 71 (12):1565-1575. doi:10.4088/JCP.09r05786blu
44. Abdelmawla AH, Langley RW, Szabadi E, Bradshaw CM (1997) Bisoprolol attenuates noradrenaline- and phenylephrine-evoked venoconstriction in man in vivo. British journal of clinical pharmacology 44 (1):61-68. doi:10.1046/j.1365-2125.1997.00624.x
45. Naso AR (2008) Optimizing patient safety by preventing combined use of intramuscular olanzapine and parenteral benzodiazepines. American journal of health-system pharmacy : AJHP : official journal of the American Society of Health-System Pharmacists 65 (12):1180-1183. doi:10.2146/ajhp070460
46. AstraZeneca (2018) SEROQUEL PRODUCT MONOGRAPH.
47. Subramanian A, Adhimoolam M, Kannan S (2018) Study of drug-Drug interactions among the hypertensive patients in a tertiary care teaching hospital. Perspectives in clinical research 9 (1):9-14. doi:10.4103/picr.PICR_145_16
48. Juarez-Cedillo T, Martinez-Hernandez C, Hernandez-Constantino A, Garcia-Cruz JC, Avalos-Mejia AM, Sanchez-Hurtado LA, Islas Perez V, Hansten PD (2016) Clinical Weighting of Drug-Drug Interactions in Hospitalized Elderly. Basic & clinical pharmacology & toxicology 118 (4):298-305. doi:10.1111/bcpt.12495
49. Katritsis D, Camm AJ (2003) Quinolones: cardioprotective or cardiotoxic. Pacing and clinical electrophysiology : PACE 26 (12):2317-2320. doi:10.1111/j.1540-8159.2003.00367.x
50. Drew BJ, Ackerman MJ, Funk M, Gibler WB, Kligfield P, Menon V, Philippides GJ, Roden DM, Zareba W, American Heart Association Acute Cardiac Care Committee of the Council on Clinical Cardiology tCoCN, the American College of Cardiology F (2010) Prevention of torsade de pointes in hospital settings: a scientific statement from the American Heart Association and the American College of Cardiology Foundation. Circulation 121 (8):1047-1060. doi:10.1161/CIRCULATIONAHA.109.192704
51. GmbH BII (2016) JARDIANCE, SUMMARY OF PRODUCT CHARCTERISTICS
52. Sanofi (2016) Lovenox, Summary of product characteristics.
53. Virtanen K, Janne J, Frick MH (1982) Response of blood pressure and plasma norepinephrine to propranolol, metoprolol and clonidine during isometric and dynamic exercise in hypertensive patients. European journal of clinical pharmacology 21 (4):275-279. doi:10.1007/bf00637613
54. Lilly E (2011) Zyprexa, Summary of product characteristics.
55. Gudmundsdottir H, Aksnes H, Heldal K, Krogh A, Froyshov S, Rudberg N, Os I (2006) Metformin and antihypertensive therapy with drugs blocking the renin angiotensin system, a cause of concern? Clinical nephrology 66 (5):380-385. doi:10.5414/cnp66380

56. Hasnain M, Vieweg WV, Howland RH, Kogut C, Breden Crouse EL, Koneru JN, Hancox JC, Digby GC, Baranchuk A, Deshmukh A, Pandurangi AK (2014) Quetiapine, QTc interval prolongation, and torsade de pointes: a review of case reports. Therapeutic advances in psychopharmacology 4 (3):130-138. doi:10.1177/2045125313510194
57. Brandt J, Leong C (2017) Benzodiazepines and Z-Drugs: An Updated Review of Major Adverse Outcomes Reported on in Epidemiologic Research. Drugs in R&D 17 (4):493-507. doi:10.1007/s40268-017-0207-7
58. Hundal RS, Petersen KF, Mayerson AB, Randhawa PS, Inzucchi S, Shoelson SE, Shulman GI (2002) Mechanism by which high-dose aspirin improves glucose metabolism in type 2 diabetes. The Journal of clinical investigation 109 (10):1321-1326. doi:10.1172/JCI14955
59. Chang SM, Granger CB, Johansson PA, Kosolcharoen P, McMurray JJ, Michelson EL, Murray DR, Olofsson B, Pfeffer MA, Solomon SD, Swedberg K, Yusuf S, Dunlap ME, Investigators C (2010) Efficacy and safety of angiotensin receptor blockade are not modified by aspirin in patients with chronic heart failure: a cohort study from the Candesartan in Heart failure--Assessment of Reduction in Mortality and morbidity (CHARM) programme. European journal of heart failure 12 (7):738-745. doi:10.1093/eurjhf/hfq065
60. Greece B (2002) Kinzalkomb (biperiden), Summary of product characteristics.
61. Lilly P- (2011) Inotrex, Summary of product characteristics.
62. Jabbal S, Anderson W, Short P, Morrison A, Manoharan A, Lipworth BJ (2017) Cardiopulmonary interactions with beta-blockers and inhaled therapy in COPD. QJM: An International Journal of Medicine 110 (12):785-792. doi:10.1093/qjmed/hcx155
63. Noor S, Ismail M, Ali Z (2019) Potential drug-drug interactions among pneumonia patients: do these matter in clinical perspectives? BMC Pharmacology and Toxicology 20 (1):45. doi:10.1186/s40360-019-0325-7
64. Fitzmaurice MG, Wong A, Akerberg H, Avramovska S, Smithburger PL, Buckley MS, Kane-Gill SL (2019) Evaluation of Potential Drug-Drug Interactions in Adults in the Intensive Care Unit: A Systematic Review and Meta-Analysis. Drug safety 42 (9):1035-1044. doi:10.1007/s40264-019-00829-y
65. Divoll M, Greenblatt DJ, Lacasse Y, Shader RI (1981) Benzodiazepine overdosage: Plasma concentrations and clinical outcome. Psychopharmacology 73 (4):381-383. doi:10.1007/bf00426470
66. Kitamura N, Takahashi Y, Yamadate S, Asai S (2007) Angiotensin II receptor blockers decreased blood glucose levels: a longitudinal survey using data from electronic medical records. Cardiovasc Diabetol 6:26-26. doi:10.1186/1475-2840-6-26
67. Elshimy G, Techathaveewat P, Alsayed M, Jyothinagaram S, Correa R (2019) Simple Reason for Hypoglycemia: ACE Inhibitor-induced Severe Recurrent Hypoglycemia in a Nondiabetic Patient. Cureus 11 (8):e5449-e5449. doi:10.7759/cureus.5449
68. Yahyavi-Firouz-Abadi N, Tahsili-Fahadan P, Ghahremani MH, Dehpour AR (2007) Melatonin enhances the rewarding properties of morphine: involvement of the nitric oxidergic pathway. Journal of pineal research 42 (4):323-329. doi:10.1111/j.1600-079X.2007.00422.x
69. Koller EA, Weber J, Doraiswamy PM, Schneider BS (2004) A survey of reports of quetiapine-associated hyperglycemia and diabetes mellitus. The Journal of clinical psychiatry 65 (6):857-863. doi:10.4088/jcp.v65n0619
